# Lipid polymer hybrid nanoparticles: a custom-tailored next-generation approach for cancer therapeutics

**DOI:** 10.1186/s12943-023-01849-0

**Published:** 2023-10-03

**Authors:** Kavita R. Gajbhiye, Rajesh Salve, Mahavir Narwade, Afsana Sheikh, Prashant Kesharwani, Virendra Gajbhiye

**Affiliations:** 1 Department of Pharmaceutics, Poona College of Pharmacy, Bharati Vidyapeeth, Erandwane, Pune, 411038 India; 2 Nanobioscience, Agharkar Research Institute, Pune, 411038 India; 3 Savitribai Phule Pune University, Pune, 411007 India; 4 Department of Pharmaceutics, School of Pharmaceutical Education and Research, Jamia Hamdard, New Delhi, 110062 India; 5 Center for Global health Research, Saveetha Medical College and Hospitals, Saveetha Institute of Medical and Technical Sciences, Saveetha University, Chennai, India

**Keywords:** Lipid polymer hybrid nanoparticles, Tumor microenvironment, Polymer-based nanoparticles, Targeted drug delivery, Cancer therapy, Smart polymers, Nanomaterials

## Abstract

Lipid-based polymeric nanoparticles are the highly popular carrier systems for cancer drug therapy. But presently, detailed investigations have revealed their flaws as drug delivery carriers. Lipid polymer hybrid nanoparticles (LPHNPs) are advanced core–shell nanoconstructs with a polymeric core region enclosed by a lipidic layer, presumed to be derived from both liposomes and polymeric nanounits. This unique concept is of utmost importance as a combinable drug delivery platform in oncology due to its dual structured character. To add advantage and restrict one’s limitation by other, LPHNPs have been designed so to gain number of advantages such as stability, high loading of cargo, increased biocompatibility, rate-limiting controlled release, and elevated drug half-lives as well as therapeutic effectiveness while minimizing their drawbacks. The outer shell, in particular, can be functionalized in a variety of ways with stimuli-responsive moieties and ligands to provide intelligent holding and for active targeting of antineoplastic medicines, transport of genes, and theragnostic. This review comprehensively provides insight into recent substantial advancements in developing strategies for treating various cancer using LPHNPs. The bioactivity assessment factors have also been highlighted with a discussion of LPHNPs future clinical prospects.

**Graphical Abstract:**

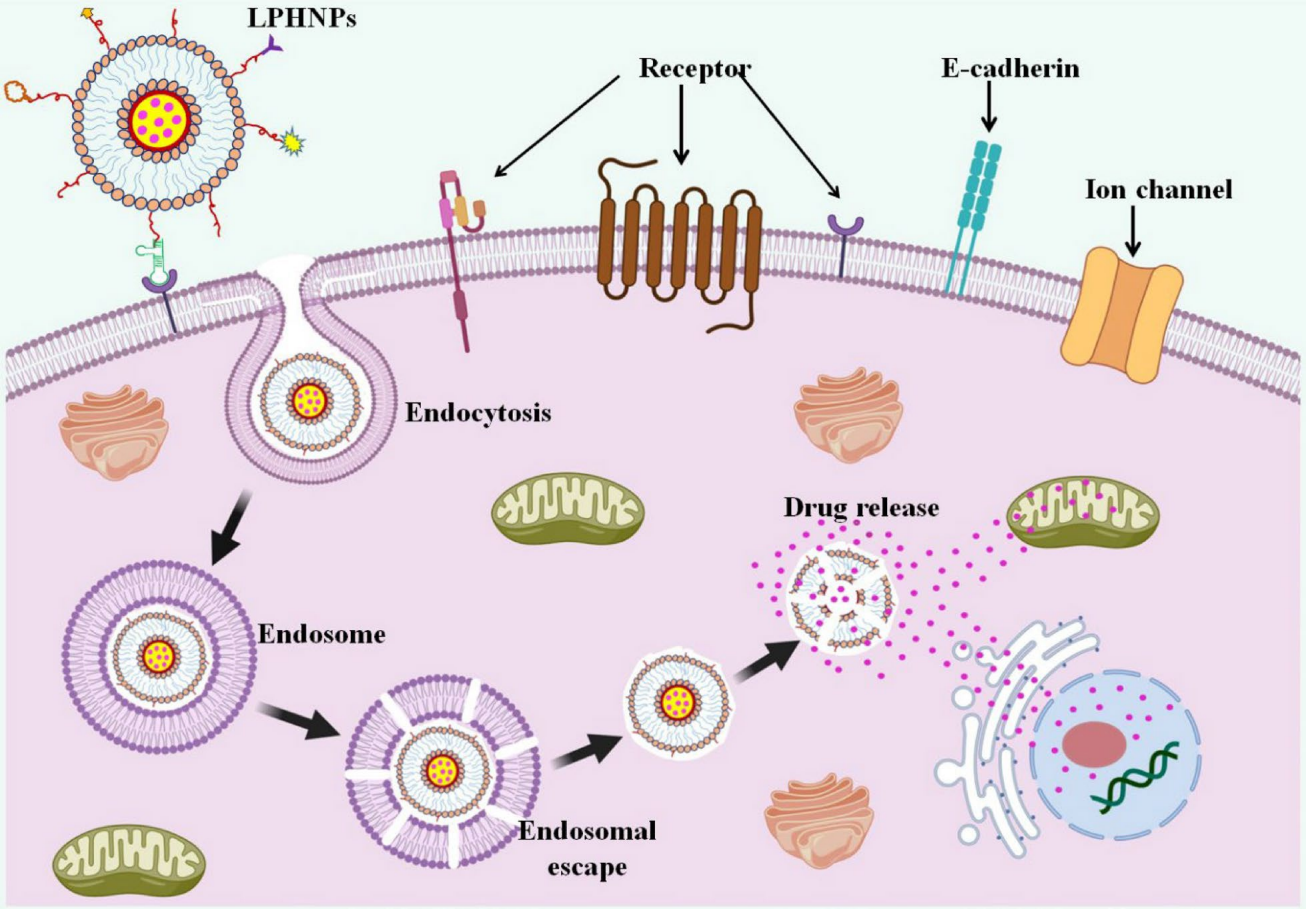

## Introduction

Cancer is the highly graded and wide spread disease of all health issues, and is the leading cause of mortality as well as morbidity around the globe [1]. The year 2018 detected more than 18 million persons with cancer with more than 9 million deaths. By 2040, the number will be doubled. According to the WHO's cancer statistics report, India's estimated cancer patient population for 2020 was around 1.3 million [2]. The annual percent change in cancer frequency rate demonstrated as rise in all types of cancer in both males and females, especially in metropolitan regions. Generally, breast, cervix, uteri, head and neck, and stomach cancers are diagnosed at the locally progressing stage [3–14]. However, few cancers, such as lung cancer, are diagnosed at the distant metastatic stage in males and females [5, 9, 15]. Cancer is responsible for over a third of all noncommunicable disease-related deaths in adults aged 30–69 years [2]. Drug resistance and metastasis in chemotherapy are major concerns in cancer therapy, making this disease even more challenging to treat. Nanoparticles (NPs)-based carrier systems have emerged as a boon for cancer theranostics. Nanocarrier systems, such as dendrimers [16–22], liposomes [23, 24], solid lipid nanoparticles [25–28], polymeric nanoparticles [29–35], micelles, niosomes [36, 37], carbon nanotubes [38–42] Quantum dots, Mesoporous silica nanoparticles (MSNPs) [43–45], Gold nanoparticles [46, 47] and self-emulsifying nano-drug delivery systems [48, 49], all have drawbacks. These drawbacks include quick drug release, drug leakage, lack of precise release, and dose-related toxicity. Instability, biocompatibility, membrane permeability, drug bioavailability, toxicity, RES absorption, pharmacokinetics, and pharmacodynamics characteristics are all factors to consider [50]. To overcome these obstacles, researchers have been working on numerous innovative drug delivery methods in order to improve therapeutic outcomes of chemotherapy by utilizing their nanostructured multifunctionality. Polymeric NPs and liposomes have exclusive benefits over other carrier systems because of their construction, composition, high structural integrity, biodegradable materials, higher biocompatibility, and controlled drug release in the biological microenvironment [51–55]. But liposomes and polymeric NPs have their own set of constraints, such as burst release and RES uptake. As a result, the formulation of various composites and combination carrier assemblies is becoming increasingly popular, as they demonstrate a more impactful and limitation-free effect in delivering medicine to the targeted spot while causing no harm to the body. Lipid polymer hybrid nanoparticles (LPHNPs) are combination of lipids and polymers which were discovered for utilizing beneficial characteristics of both. 

## Types of LPHNPs

LPHNPs can be classified on the basis of their architecture combining lipid and polymeric structure in core and shell, respectively. The section below entails various categories of LPHNPs depending upon respective attributes.

### Lipid based polymeric hybrid nanostructures

**Fig. 1:**
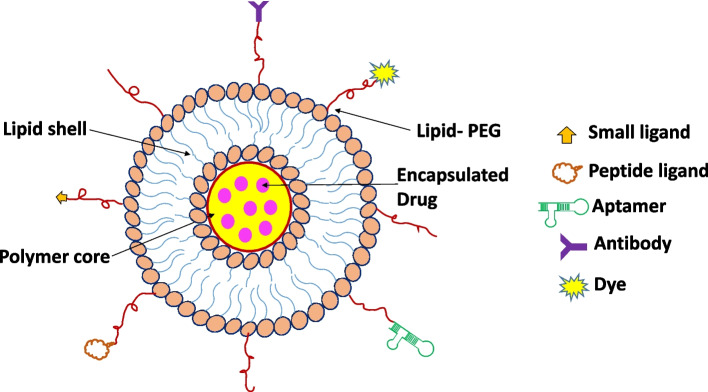
Schematic representation of multifunctional LPHNPs comprising structural components and various possible functionalities

**Fig. 2:**
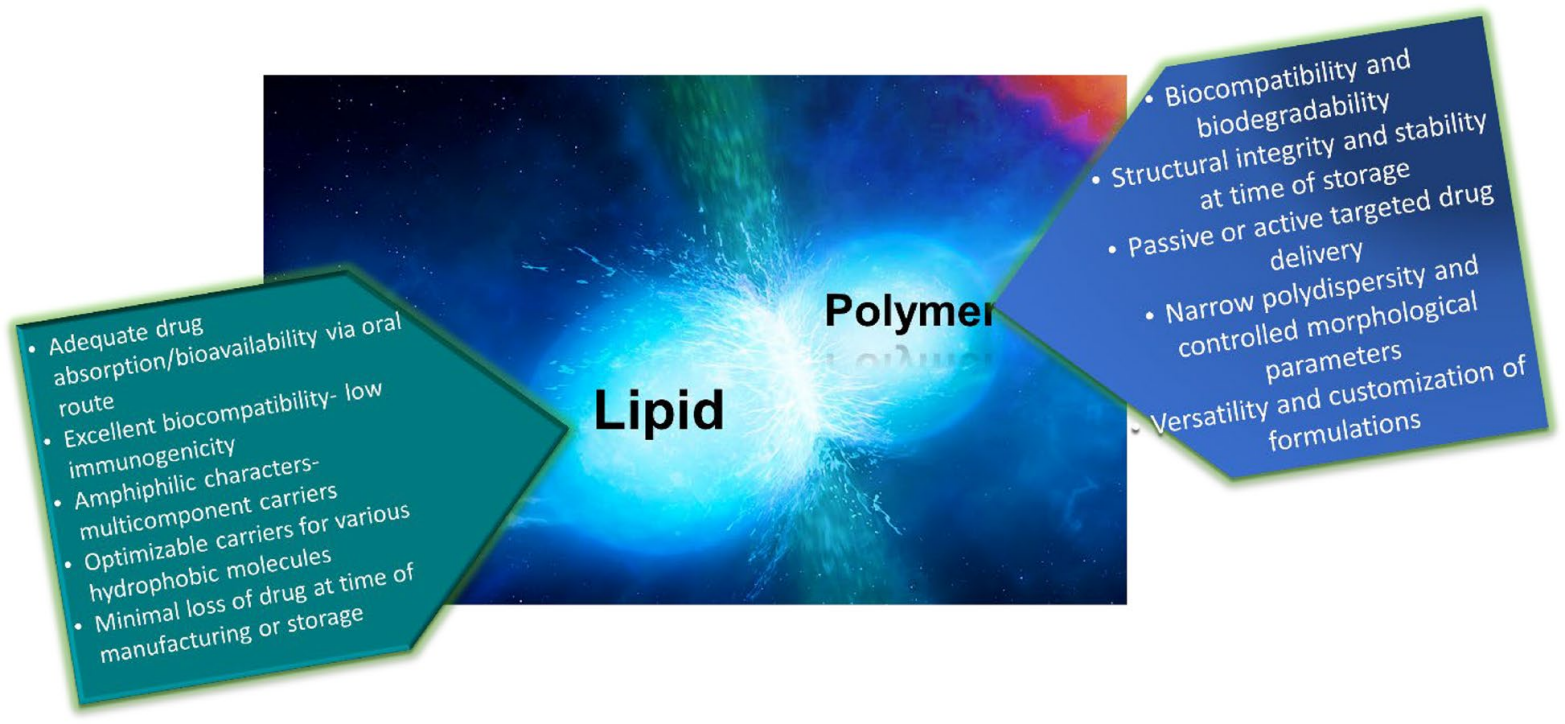
A adequacy of polymer and lipid as novel therapeutic carrier overcoming one-others limitations

In riposte to aforementioned drawbacks and adhering issues, an evolutionized foundation with effective treatment modality has been issue to lay down a delivery system based on deployment of both liposomes and polymeric nanoparticles named as lipid polymer hybrid nanoparticles (LPHNPs) [56]. The biomimetic activity of the lipid materials and mechanistic advantages of the polymeric agents influence newly developed LPHNPs system [57, 58]. LPHNPs can be synthesized by a mixture of natural/synthetic/semi-synthetic polymers and lipids that exhibit a variety of properties, including structural functionality, architectures, size, shape, exterior charge, responses to internal and external stimuli, and suitability for entrapping a large number of bioactive molecules in both core and shell positions [59–63]. For attaining biodegradability and biocompatibility, the core of the LPHNPs could be made up of polymeric biomaterial like poly lactic acid (PLA), polycaprolactone (PCL), pluronic F-68, chitosan, etc.Other than these, myristic acid, Phosphatidyl choline (PC), cholesterol, and 1,2-dipalmitoylsn-glycero-3-phosphocholine (DPPC), stearic acid, 1,2-distearoyl-sn-glycero-3-phosphoethanolamine (DSPE), soya phosphatidylcholine (SPC) and 1,2-dilauroyl-sn-glycero-3-phosphocholine (DLPC) are some of the lipids found in the lipid shell [64–69]. The assembly of LPHNPs is divided into three sections i) Polymer core- in which the drug is contained; ii) Lipid monolayer- polymeric core surrounded by lipid monolayer, which reduces drug release from the polymeric core while also protecting the core by inhibiting water transport and iii) Lipid PEG layer- in which special targeting moieties can be conjugated (Fig. 1). As a preferential application of outer PEGylated layer, LPHNPs show longer circulation/retention time by avoiding immune responses [70]. Because of the polymeric core, the LPHNPs have good structural integrity, storage stability, and controlled release qualities, while the lipid and lipid–PEG layers have advantages of great biocompatibility and bioavailability (Fig. 2).

**Fig. 3:**
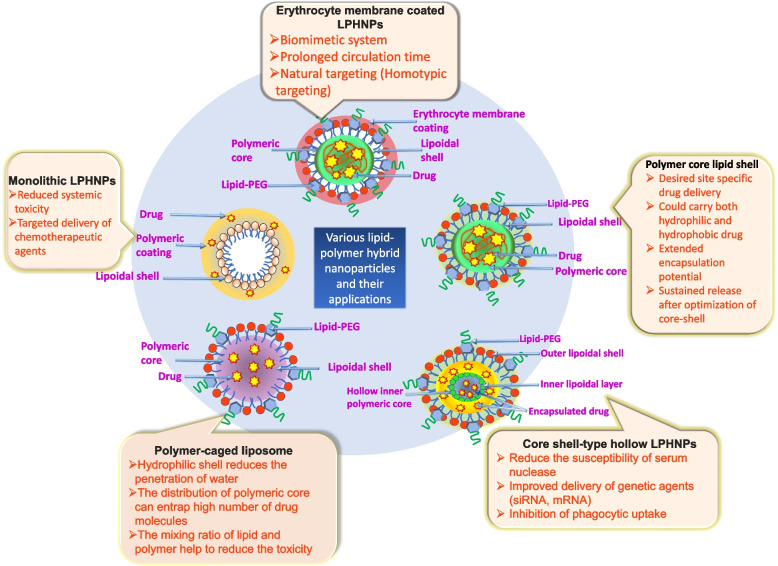
Graphical illustration of various LPHNPs and their application in drug delivery

Because of these properties, LPHNPs are very effective, convenient, and dependable drug delivery carriers [71]. Further, the pertinent ingredient the features of the used ingredients and the structural architecture of the final delivery system influence the drug release behaviour of conjugated and encapsulated payload entities. LPHNPs possessing ingredient with proficient artifacts influence a variety of release mechanisms, including erosion, diffusion, membrane fusion, endocytosis, passive and active targeting, and a tuneable response to various physical stimuli such as electricity, temperature, pH as well as biological effects such as antigens, metabolites, or enzyme [72, 73]. The LPHNPs composite construction allows for a wide range of polymer and lipid combinations (Fig. 3).

### Monolithic hybrid system

The archeological parameter of monolithic hybrid systems, also known as mixed lipid-polymer hybrid NPs, have a unique architectural dimension in which molecule of lipid are randomly scattered and create the core into which hydrophobic drugs can be loaded. Such a combination strategy of nanoparticulate system serves as colloidal vehicle for encapsulation of hydrophobic drugs. Phospholipids are an important part of the hybrid structure that forms the carrier-like structure in this case. These LPHNPs can be used to entrap extremely lipophilic drug molecules that would otherwise not be entrapped inside the polymer. The mixing ratios of the lipid and polymer can be altered and optimized during manufacturing to prevent systemic toxicity throughout the body [74].

### Polymer-core lipid-shell hybrid nano-systems

The composition of polymer-core lipid shell hybrid system comprises of a polymeric core which is surrounded by the lipid layer forming the lipid shell. The therapeutic agents are loaded inside the polymer core in such a system, while the lipid layer ensures the system's biocompatibility [75–77]. In between the lipid and polymeric core, the space is filled by the aqueous layer or water. Adopting the benefits, the release of drug was influenced by the polymer improving the stability of the lipid layer. The main disadvantage of this LPHNPs system is that hydrophobic drugs can readily be loaded, whereas encapsulating hydrophilic drugs is difficult. To overcome this, a complex of polymers and lipids can be used to enhance the loading of hydrophilic drugs. Herein, the encapsulation of hydrophobic compounds in the lipophilic lamellar junction and hydrophilic components on the bilayer are majorly influenced by the amphiphilic lipid usage. This property permits various hydrophilic and hydrophobic therapeutic substances to be entrapped and delivered at the same time [78].

### Hollow core lipid-polymer-lipid NPs

The composition of hollow core lipid-polymer lipid NPs are defined by the empty inner core whose boundaries are developed using cationic lipid layer, followed by coating with polymeric shell made of hydrophobic components further surrounded by neutrally charged outside PEG lipid layer coated by the polymer matrix. This system is made up of a hollow inner core with positively charged inner lipid layers, a middle hydrophobic polymer layer (e.g., PLGA), and a neutrally charged outside PEG lipid layer coated by the polymer matrix. The distinct layers of these NPs have crucial benefits of hiding themselves from macrophages, sustained drug release, and encapsulating anionic drugs [79–82]. The advantage of this system is that it can deliver two therapeutic agents simultaneously. For example, within the hydrophobic PLGA layer, a combination of siRNA and a synergistic small-drug molecule may be beneficial for the treatment of a variety of disorders, including multidrug-resistant malignancies [83–92].

### Biomimetic LPHNPs

Biomimetic lipid-polymer hybrid NPs, also known as cell membrane-camouflaged polymeric NPs, are created by covering the polymeric core using the cellular membrane. The NPs are then encased in RBCs, platelets, leucocyte, having the capacity to remain in the systemic circulation for a longer duration of time so protect them from macrophage uptake. RBC has a more robust lipid barrier against drug release; thus, it may slowly release drugs while leucocyte camouflaged system enable targeting of cancer cells as they escort them to reach the site of inflammation Apart from the benefits, the major hurdle upon utilization of such an approach is the availability of distinct antigens on the erythrocytic surface, which may cause immunogenic effect upon blood transfusion making it unsuitable for patients with different blood groups [58, 93–96].

### Polymer caged liposome NPs

The discovery of polymer encapsulating liposomes might have occurred to overcome the challenges like susceptibility of liposomes to enzymes, pH and immune system of the body as well as different physiological conditions. It leads to premature leakage of the cargo and lowers therapeutic efficiency. The polymer caged liposomes design of LPHNPs involves a hydrophilic core with payload and outer covering of a polymeric structure. Such structural organization provides stability to the nanoparticles, avoiding drug leakage and decreasing its release in the systemic circulation. To enable controlled drug release, sometimes, the outer covering of the polymer may make use of pH or protease sensitivity [97, 98]. This can protect the cargo and provide a sustainable payload release at the target site. Occasionally, polymer cages may easily dissociate from the liposome surface and return to an unstable state, which is one of the major drawbacks of these NPs. Further, RES can readily capture polymer caged liposomes, and their uptake may be lowered due to the absence of a lipid layer over their surface [10, 97–100].

### Advantages of LPHNPs

The capacity of LPHNPs to solubilize hydrophobic drugs in the system, the capacity to combine various types of drugs (hydrophilic and lipophilic) simultaneously, and the capacity to reduce the exposure of host organs to potentially harmful drugs are all major advantages of LPHNPs. The use of biomimetic lipids or PEG in LPHNPs increases the stability and circulation time of the vehicle during drug delivery by minimizing drug release and reducing RES interactions. The unique properties of the LPHNPs structure make them promising carriers for dual drug delivery and nucleic acid therapeutics delivery. Other advantages include permitting the carrier surface to be modified and functionalized by a variety of chemical moieties. The surface modifications of LPHNPs were made to meet two critical goals- (I) active drug targeting (ligands can be linked to a carrier system that is recognized by overexpressed receptors on the tumorous cells being targeted) and (II) modification can be used to give the composite a stimuli-sensitive or "intelligent/smart" quality, allowing the cargo to release drugs on-demand only when certain stimuli are present [101, 102]. Recently, dual-responsive delivery systems have been devised. This demonstrates a combination of reactions not only at the diseased site but also in the NPs manufacturing process, NPs carrying routes, and cellular compartments all at the same time. The dual and multi-stimulus responsive LPHNPs provide better control over programmed site-specific drug delivery, resulting in fantastic anti-cancer efficacy in vitro and in vivo [103–105]. Active targeting is a key feature of nanomedicine in cancer therapy for enhancing drug reach-ability to the intended region, which leads to therapeutic success and reduces adverse effects [16, 106–110]. Various ligands may be linked to the surface of LPHNPs due to their structural specificity and multi-functionality, allowing for therapeutic targeting of tumorous sites [111–116] Antibodies [117, 118], peptides [119, 120] folate [121–124], transferrin [125–128] and aptamers [129–134] are some of the ligands employed for targeting LPHNPs. This nanocarrier system may also be proved effective for the oral route of drug administration against life-threatening diseases [135–137].

## Synthesis methodologies of LPHNPs

Various distinct methodologies with different processes appear to have engaged in the formulation process of LPHNPs. In one-step procedures, the precipitation of polymeric network is enabled through homogenization of the aqueous phase with the organic phase, self-assembling to form single layer unit to surround the core. At the same time, PEGylated lipids self-assemble in a systematic fashion, with the lipid moiety arranged on the polymer core's surface layer and the PEG chain providing an exterior extension for attachment of various functional moieties [50, 138, 139]. The two-step method of LPHNPs preparation shows a disputable mechanism of initial structureal bilayer formation with good adhesion to the core. This leads to the subsequent disintegration process of the initial bilayer of polymer and lipid chains due to the hydrophobic interaction. In terms of hydrophobic, van der Waal, and electrostatic interaction phenomena, the production of composite is thermodynamically advantageous [78, 140–143]. Major preparation techniques are discussed below in brief.

### Synthesis of monolithic hybrid system

This type of NPs can be synthesized by simultaneously dissolving the entire component drug, lipid, and polymer into the organic phase under slow heating. Further, the resultant mixture can be heated to remove the organic phase. The aqueous phase can be added to the previously obtained mixture, followed by sonication and purification by centrifugation. Here, the drug is encapsulated inside the lipid and scattered throughout the polymer. This method can encapsulate and safely deliver the lipophilic drug [144].

### Synthesis of polymer-core lipid-shell NPs

These LPHNPs can be synthesized by multiple methods like nanoprecipitation, emulsification–solvent evaporation, etc. During the synthesis, a polymer dissolved in the organic phase can be added into lipid (aqueous medium), followed by sonication and centrifugation. In such synthesis, the entrapment of the drug can be done by dissolving the drug into the organic phase [75, 78, 145]. The drug-to-polymer ratio is a significant aspect in the synthesis of core–shell LPHNPs as an incorrect parameter causes formation of lumps, leading to the failure of the process.

### Synthesis of hollow core lipid-polymer-lipid NPs

These NPs could be synthesized by the double emulsion and self-assembly method. Here, the lipid facing the core could be mixed with the polymer containing organic phase. Therapeutic molecules dissolved in the aqueous phase can be further added to the organic phase dropwise under sonication, which forms the first emulsion. Further, this emulsified mixture could be incorporated into the solution containing PEG and shell-forming lipid under sonication, forming the second emulsion [146]. To create these hybrid structures, certain factors are required to kept in mind such as the density of PEG network, polymer's molecular weight and charge of the lipidic structure [84].

### Synthesis of biomimetic LPHNPs

Synthesis of biomimetic LPHNPs includes membrane derived from the RBCs, leucocytes, platelets and the nanoparticles carrying the vesicular structure. In brief, the polymeric NPs are firstly synthesized and suspended into the aqueous phase. Then, membrane is suspended and lysed inside PBS separately. The lysed cells are collected by centrifugation followed by sonication to form vesicles. Further, the vesicles can be added to the polymeric NPs solution to form a cell membrane coating on the outer side [147–150].

### Synthesis of polymer-caged liposome NPs

The extensive structure of the polymer caged liposomes is a result of its decoration with the hydrophobic group anchored polymers and water loving polymers functionalizing the structural unit of liposomes [151–155]. Liposomes caged with a single polymer layer can be made by dropping polymer solution into a liposome dispersion that has already been prepared. Furthermore, to extend the stability and inhibit the polymer dissociation, liposomes could be functionalized with cross-linked polymers [156–160]. However, this might occasionally come at the sacrifice of controlled payload release [97, 99, 161–163].

## Application of LPHNPs for treatment of the different carcinomatous conditions

**Fig. 4:**
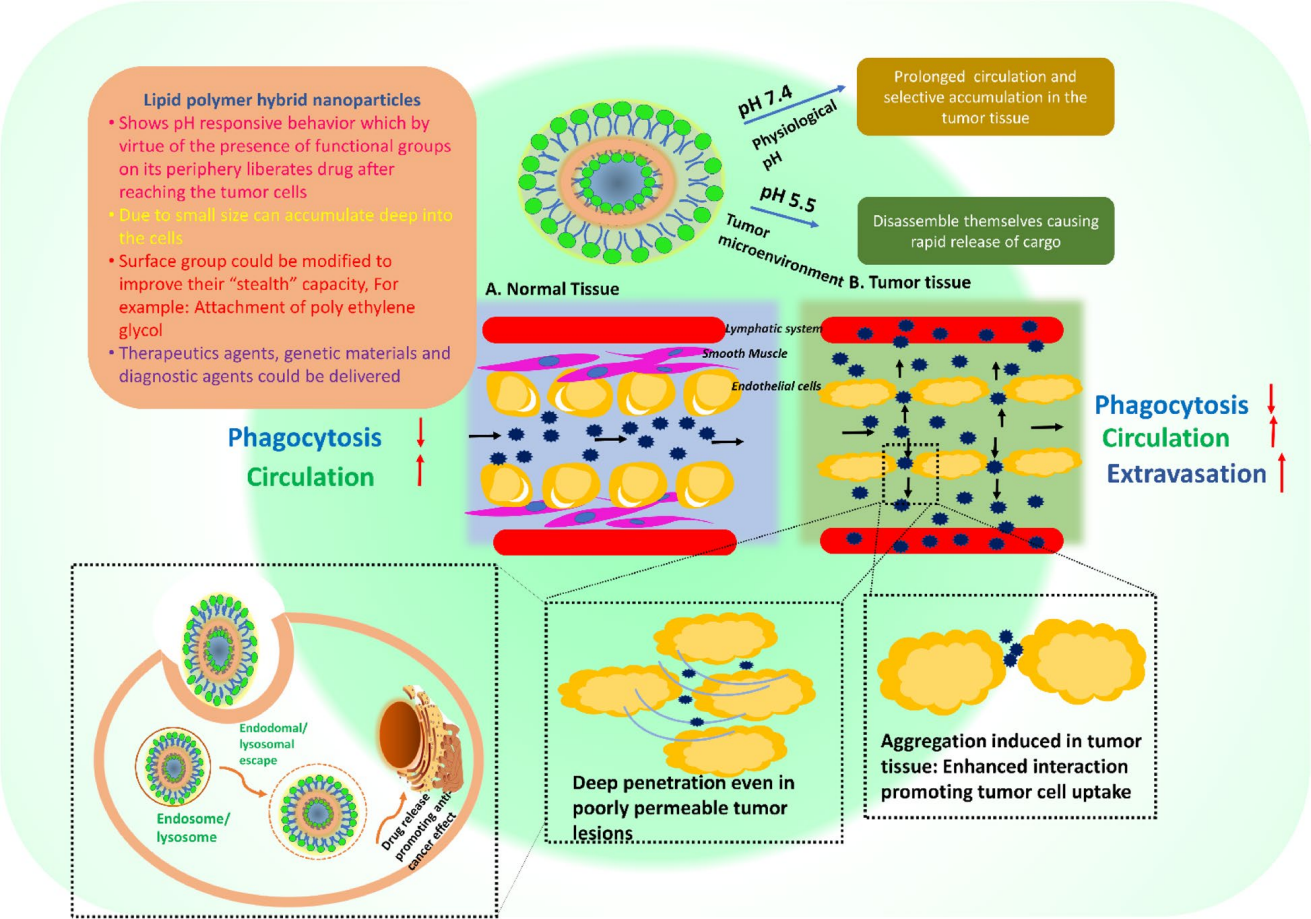
The interaction pattern of LPHNPs with cancer cells. At physiological pH, the LPHNPs show a delayed release while after recognizing the tumor cells, rapid release of the drug

**Fig. 5:**
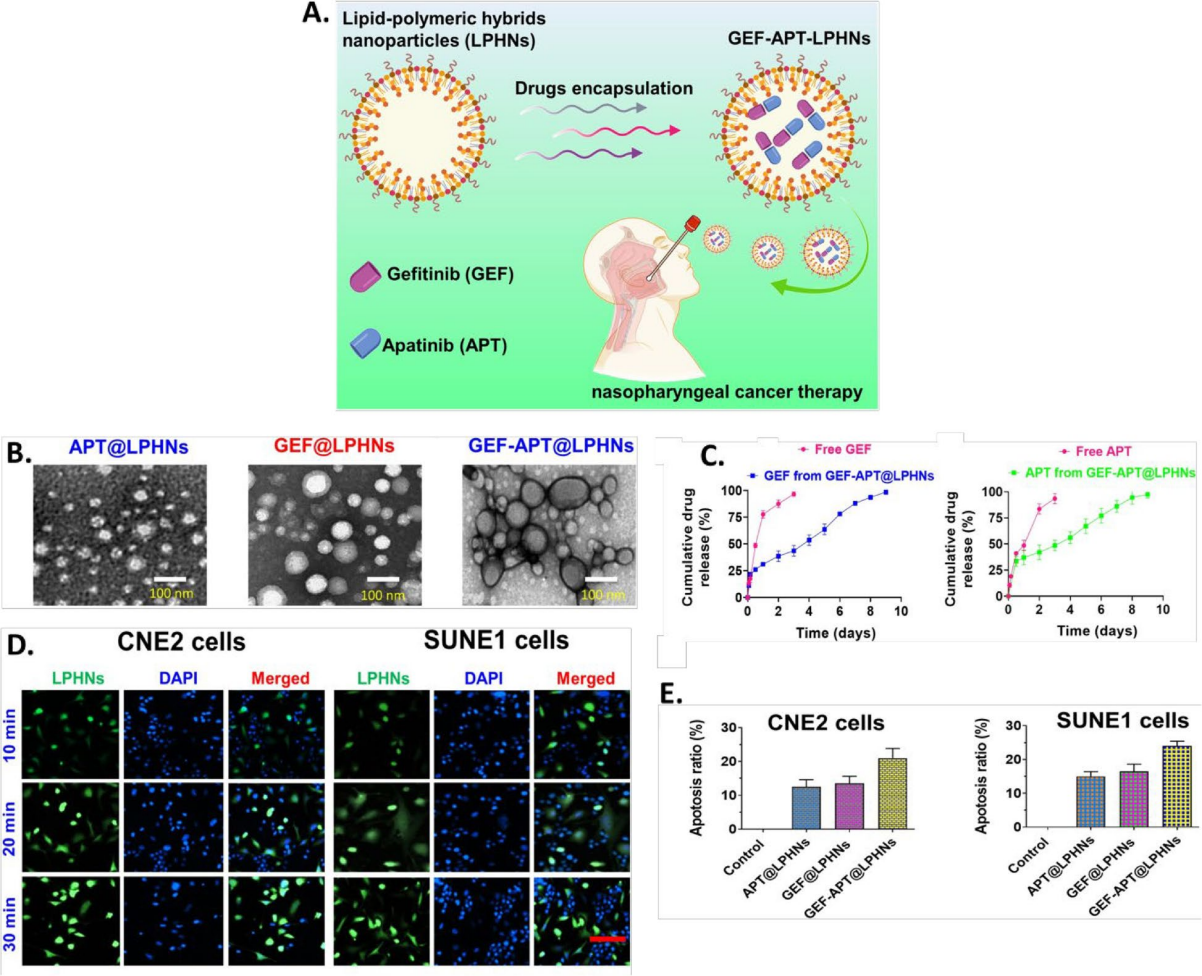
Illustration of dual drug loaded (Afatinib and geftinib) in core-shelled construct of lipid-polymeric nanoparticles in nasopharyngeal cancer therapy (**A**), Transmission electron microscopic images of apatinib, geftinib and dual drug loaded hydrid nanosconstructs (from left to right) (**B**), In vitro drug release study of apatinib and geftinib from nanostructures loaded with both drugs (**C**), Fluorescent uptake and cell apoptosis study with different preparations suggesting dual drug loaded system is highly essential in treatment of nasopharyngeal cancer (**D**), Cellular uptake efficacy of the different formulations of GEF-APT@LPHNs examined by flow cytometer analysis (**E**). Reproduced with permission from Yu et al. [165]. Copyright (2021) Elsevier

In diverse types of carcinomas, LPHNPs are effective in delivering a single medicine, a combination of medicines, or even multidrug delivery. The best choice of LPHNPs for cancer therapeutic development mainly depends on the characteristic of the drug molecule (hydrophilic or hydrophobic or nucleic acid (siRNA, miRNA). For more prominent delivery LPHNPs can be ligated with the cancer cell-specific ligand molecules, which are eagerly engulfed by the cancer cell. The combined potential of encapsulating and controlled release pattern of cargo upon reaching the cancer cells makes them a fascinating system for in vitro and in vivo investigations in various malignant conditions (Fig. 4) [164]. Several studies have indicated the benefits of employing LPHNPs for the treatment of cancers of the breast, lung, liver, prostate, skin, blood, bone, brain, and other organs [55]. The application has also been widened in treatment of nasopharyngeal cancer as well. Working on it, the researchers Yu and Zhang developed gefitinib and apatinib loaded lipid polymer hybrid nanostructures which showed prolonged release, better uptake and enhanced cell cytotoxicity proving efficacy against such rare cancerous conditions [165] (Fig. 5). With a view to reduce various cancer cells, conferone and methotrexate loaded lipid polymer hybrid nanoparticles were developed to enhance the cancer cells internalization, induce apoptosis and prolong the anti-cancer effect [166]. A few of the examples are discussed in it.

### LPHNPs for breast cancer therapy

Breast neoplasm is one of the common cancers and is foremost reason for death in females globally. Approximately, among all types of cancers, breast tumor holds 10% of share in females worldwide, holding it the 2^nd^ fewest prevalent kind of non-skin cancer and the 5^th^ most predominant reason for cancer mortality [167–170]. It is divided into three groups depending on the existence or nonexistence of a molecular target for various receptors, such as estrogen receptor (ER), progesterone receptors (PR), human epidermal growth factor 2 (ERBB2 or formerly called HER-2), and hormone receptor ERBB2 negative. The ERBB2 negative and triple-negative cancers are kind of cancer lacking all three typical molecular markers. The treatment schemes varied according to the molecular subtype [171, 172]. An interdisciplinary course of therapy is available till date in the treatment of breast cancer which considers both systemic therapy and locoregional therapy comprising radiation and surgical resurrection. The conventional treatment approach relies upon inhibitors blocking BRCA mutation (polymerase inhibitors), anti-HER2 treatment, hormonal therapy while newly developed approach incorporates the targeted therapy with ligand identification to attach with the over-expressed receptors [173–176]. Based on tumor biology and primary treatment outcomes, the future therapeutic scope in breast cancer treatment anticipates aims such as the creation of tailored medicine and treatment reduction [177, 178]. LPHNPs have been extensively explored for the delivery of chemotherapeutics moieties to treat breast cancers. Few examples are discussed in this heading and Table 1 summarizes the LPHNPs-based therapeutics for breast cancer therapy.

**Table 1:** LPHNPs in breast cancer therapy

Sr. No	Lipid component	Polymer component	Targeting moiety	Drug	In vitro	In vivo	Ref
1	DSPE-PEG-2000	PLGA	_	DTX	MDA-MB-231	Female Balb/c mice	[179]
2	SPC and DSPE-PEG	PLGA	_	GEM	_	_	[139]
3	SPC and DSPE-PEG-2000	PLGA	_	GEM	MCF-7 and MDA-MB-231	Sprague–Dawley rats	[180]
4	Lipoid S100	PLGA	_	MTX	MDA-MB-231	_	[50]
5	lecithin with DSPE-PEG-2000	PLGA	_	SFN	MDA-MB-231	_	[181]
6	Soybean phospholipid and DEPE-PEG-2000	PLGA	_	Emodin	MCF-7/ADR	_	[182]
7	Dioleoylphosphoethanolamine (DOPE), oleic acid and DSPE-PEG-2000	PLGA	_	DTX	MCF-7 and MDA-MB-231	Balb/C female mice	[183]
8	lecithin and DSPE-PEG	PLGA	_	CPT	MT2 mouse breast cancer cells	_	[61]
9	Tristearin, stearic acid	Pluronic-F68	_	DOX and Elacridar	MDA-MB-435/LCC6/MDR1	_	[184]
10	Dioleoylphosphatidic acid	PLGA-PEG	_	microRNA-222 and PTX	MDA-MB-231	_	[185]
11	Soya lecithin and DSPE-PEG	Pluronic-F68	_	Mycophenolate and Quercetin	MCF-7	Sprague Dawley rats	[145]
12	DSPE-PEG-3000, stearyl amine,	PLA	L-fructose	MTX and beta-carotene	MCF-7	female Wistar rats	[186]
13	Phospholipid S100 and DSPE-PEG-2000	PCL	Fucose	MTX and aceclofenac	MCF-7 and MDA-MB-231	Female BALB/c mice	[187]
14	Egg lecithin, DSPE-PEG-2000	PLGA	cRGD	HCPT	MDA-MB-435 s	_	[188]
15	Soya lecithin and DSPE-PEG-2000	PLGA	iRGD	Isoliquiritigenin	MCF-7, MDA-MB231, and 4T1 cells	female nude mice	[189]
16	DSPE-PEG-2000 and DSPE-PEG-2000-FA	PLGA	Folic acid	Indocyanine green and Cisplatin	MCF-7	_	[190]
17	N,N-bis(2-hydroxyethyl)-N-methyl-N-(2-cholesteryloxycarbonyl aminoethyl	PLA	_	Polo-like kinase 1 siRNA	BT747	BALB/c-nu nude mice	[191]
18	DSPE-PEG-2000	poly (β-amino ester), PBAE	Folic acid	Docetaxel	4T1	BALB/c	[192]

#### Single drug delivery

**Fig. 6:**
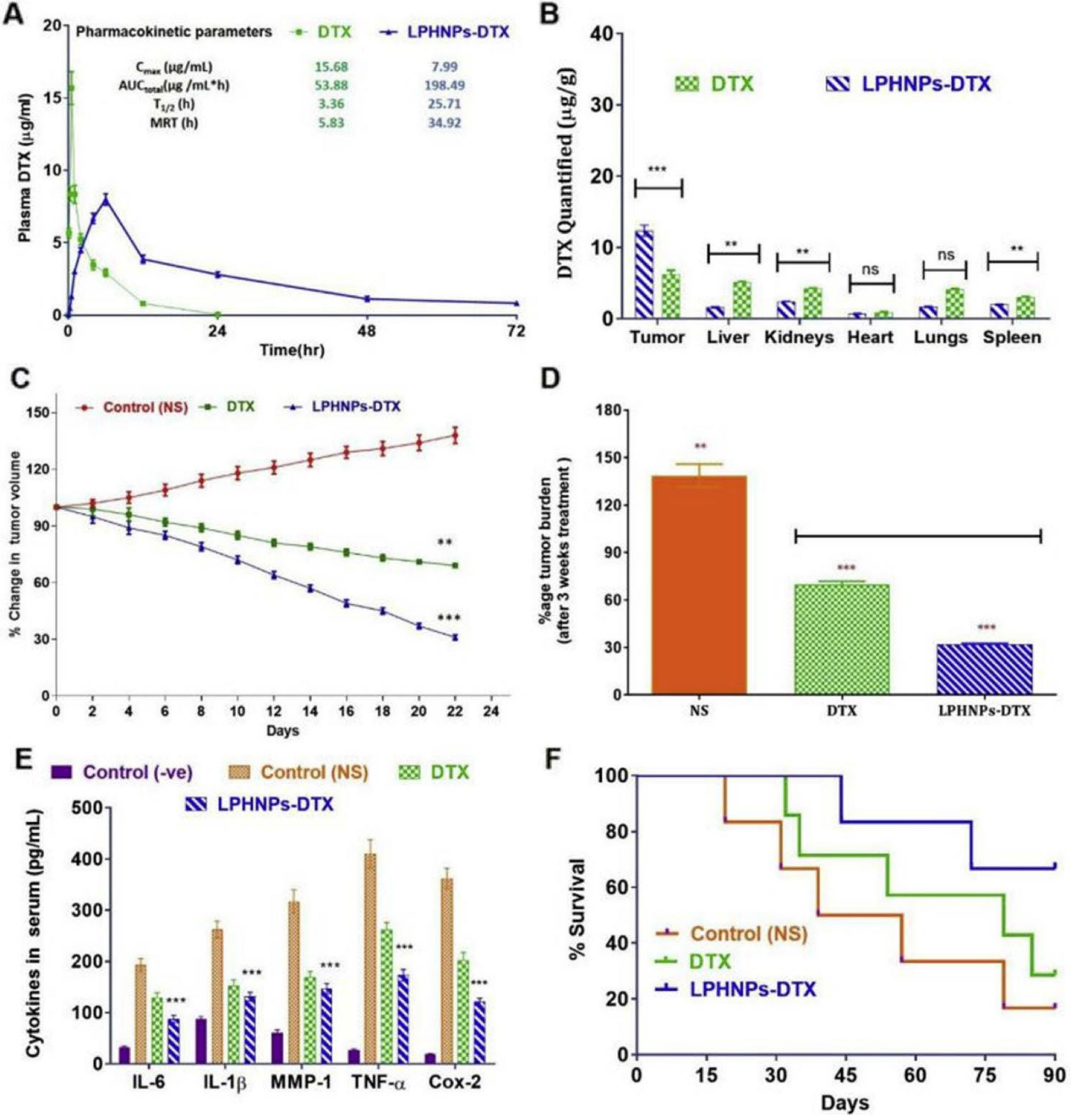
Representation of various efficacious parameters such as biodistribution, pharmacokinetics, anti-tumor efficacy, concentration of cytokines and percentage survival of animals. (**A**) Depicts the plasma concentration of docetaxel after single i.v dose, (**B**) biodistribution profile of DTX, (**C**) Assessment of in-vivo anti-tumor study, ﻿(**D**) Estimation to tumor burden, ﻿(**E**) level of cytokines after treatment, and (**F**) percentage survival as evaluated by Kaplan–Meier survival curve. Reproduced with permission from Jadon et al. [179]. Copyright (2019) Elsevier

This section discusses examples wherein a single drug is delivered via LPHNPs for breast cancer therapy. Docetaxel (DTX) is a potent anti-cancer drug from the taxane family. Nonetheless, due to their low solubility in water limits its wide application and thus, is currently delivered with Tween 80® and ethyl alcohol which has side effects like neurotoxicity, hepatotoxicity, musculoskeletal toxicity, neutropenia, hypersensitivity reactions etc. Hence, LPHNPs were employed for DTX delivery with minimum side effects. The nanoprecipitation (single-step) approach was employed to produce LPHNPs encapsulating DTX, which were evaluated for therapeutic efficacy against breast cancer. The DTX-LPHNPs revealed a pH-dependent drug release pattern in various PBS pH 5.5, 6.8, and 7.4. Dissociation of lipid (DSPE) in the early period was responsible for the cargoes burst release with simultaneous sustained release thereafter. The DTX-LPHNPs showed a greater cytotoxic effect against MDA-MB-231 cells at a 0.05-20 µg/mL concentration range. Further, DTX-LPHNPs showed higher early and late apoptosis (11.29 and 18%, respectively) than cells treated with free DTX (3.20 and 7.3%, respectively). In vivo animal experiments in a breast cancer model revealed that DTX-LPHNPs have a longer half-life and a longer mean retention time (MRT) (5–6 times) than free DTX. The presence of DSPE over the polymer increased the circulation time of the LPHNPs. The biodistribution study in the tumor-induced model revealed that after a single dose of DTX-LPHNPs, a significantly higher amount of DTX was perceived in the cancer cells as compared to free DTX (Fig. 6). The antitumor effect of DTX-LPHNPs showed a 31.9% reduction in tumor volume which was significantly better (*p* < 0.001) than free DTX (69.85%) (Fig. 6). The reduction in cytokines involved in pro-inflammatory angiogenesis was also observed for DTX-LPHNPs as compared to free DTX and control group. Overall, the DTX-LPHNPs study showed promising results in terms of progressive therapeutic participation in the battle against breast cancer [179].

**Fig. 7:**
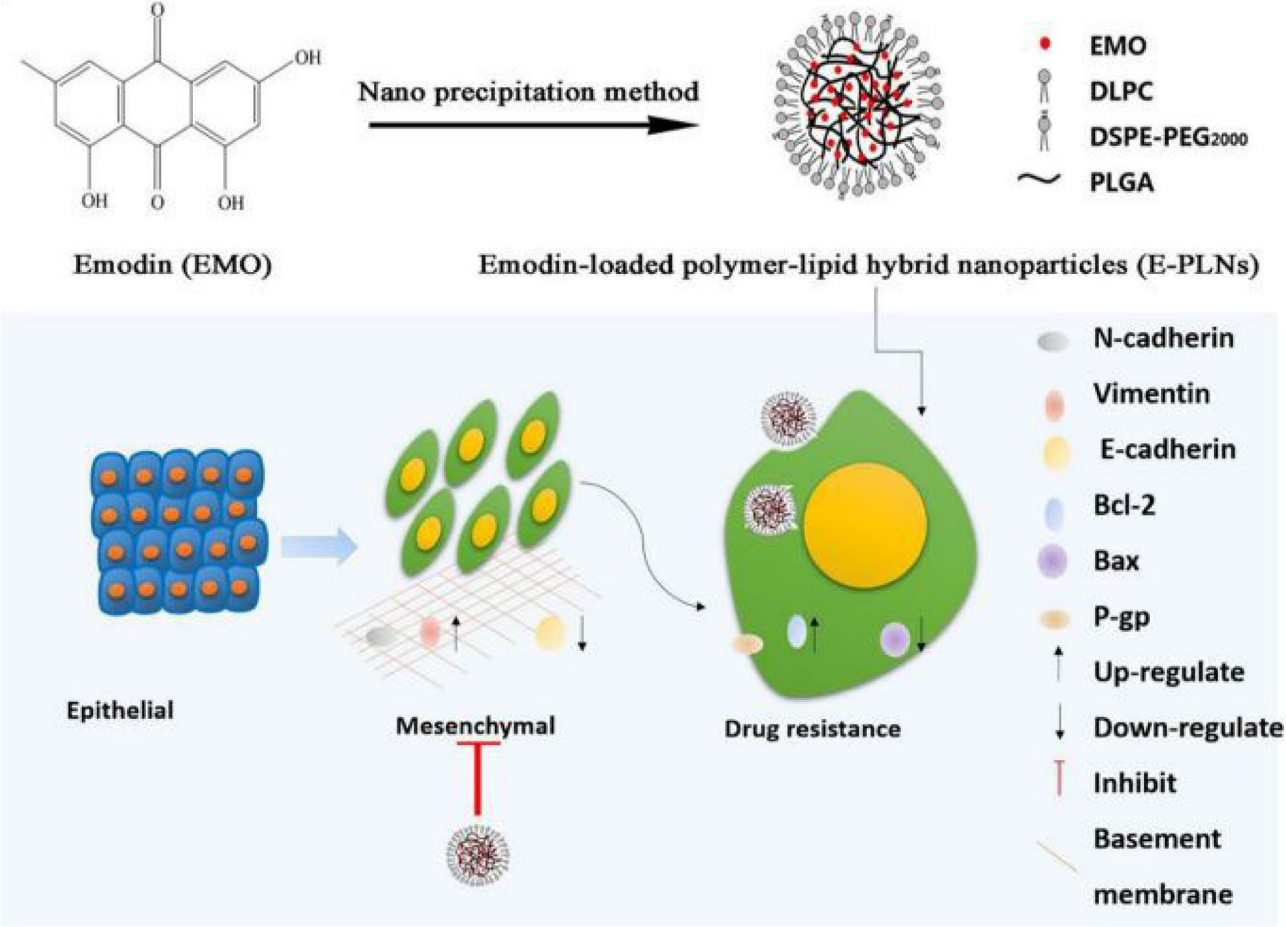
Graphical representation of synthesis of Emodin- loaded polymer-lipid hybrid nanoparticles (E-PLNs) and their effect on epithelial-mesenchymal transition of breast cancer cells. This figure is reproduced under Creative Commons Attribution 4.0 International License [182]

The modified one-step nanoprecipitation approach was used to create LPHNPs for the delivery of both hydrophilic (DOX.HCL) and hydrophobic doxorubicin (DOX) for the controlled administration of medicine to treat breast cancer. The LPHNPs revealed a time and dosage-dependent cellular absorption on MDA-MB-231 cells and PC3 cells (human prostate cancer cells) through the endocytosis route. Compared to control and free DOX solution, DOX base-loaded LPHNPs had a greater antiproliferative impact (in both cell lines) at 200 µg/mL concentration. After 48 h, the DOX-base LPHNPs formulation at 200 µg/mL had a viable cell count of less than 20%, whereas the free DOX solution had a 35–40% viable cell count. The underlining reason for such effectiveness could be due to hydrophobic-hydrophobic interaction between LPHNPs components and DOX. Thus, it could be estimated that LPHNPs could be a ground breaking approach in mediating a potential therapeutic response against breast carcinoma [193]. LPHNPs were also used to overcome small half-life and rapid inactivation of highly hydrophilic drug gemcitabine hydrochloride (GEM). Yalcin et al. (2018) optimized the synthesis of GEM-loaded LPHNPs by a central composite design approach. The different combinations of polymer (PLGA), DSPE-PEG and lipid (SPC) were investigated for LPHNPs synthesis. The high entrapment of the GEM (45.2) in the NPs was due to the presence of a higher amount of PLGA and their hydrophilic nature. The higher drug release (60.1%) was observed for the same formulation [139]. Similarly, GEM.HCl-loaded LPHNPs were utilized to improve chemotherapeutic responsiveness against breast cancer. The internalization assay of coumarin-6 loaded LPHNPs and blank LPHNPs showed higher accumulation of NPs in breast cancer cells. The cytotoxicity results suggested that GEM-loaded LPHNPs significantly reduced cell viability and lower IC_50_ value than free GEM (GEM solution and Gemko®) analyzed on breast cancer cell line. The IC_50_ values of Gemko® were reported to be 2.29 and 1.96 µM, respectively. Similarly, the IC_50_ values of GEM-loaded LPHNPs were reported to be 0.40 and 0.38 µM, respectively, indicating higher potency of GEM-loaded LPHNPs. The high cytotoxicity of GEM-loaded LPHNPs was due to the effective internalization of NPs via lipid shell interaction with cancer cells. Further, the GEM-loaded LPHNPs outperformed the marketed product Gemko® in the in vivo pharmacokinetic testing in Sprague–Dawley rats. They found that the GEM-loaded LPHNPs had higher bioavailability. The presence of a protective layer of polymer and lipid supported 4.2 times longer half-life of GEM-loaded LPHNPs formulation in comparison to commercial GEM. The outcomes entitled LPHNPs as excellent vehicle for the delivery of GEM inducing anti-cancer effect [180]. A factorial designing methodology was used with Design-Expert® 7.0.0 software to prepare MTX-loaded LPHNPs. A three-factorial, three-level Box-Behnken statistical design and 15 trials were made to optimize the LPHNPs. The synthesis showed that the size of LPHNPs increased with PLGA concentration and drug entrapment efficiency increased with both polymer and lipid concentration. The reverse dialysis technique was used to ascertain the drug release pattern of produced LPHNPs in PBS under physiological circumstances. The release pattern of the LPHNPs demonstrated an early burst release showing an initial burst of 40% within 2 h, followed by sustained release of cargo. The antiproliferative activity was tested using ATP activity-based luminescence assay in the MDA-MB-231 and PC3 cell lines. A time and dose dependent anti-proliferative effect was observed in cells treated with MTX-loaded LPHNPs and plain MTX. Compared to plain MTX solution, LPHNPs loaded with MTX demonstrated greater growth inhibition efficiency in the MDA-MB-231 as compared to the PC3 cell line. Overall, this study showed that the usage of an MTX-loaded delivery system is preferable for treating cancer [50]. In addition, microfluidic manufacturing has also been tried for the development of anti-cancer drug sorafenib (SFN)-loaded LPHNPs. These LPHNPs were developed following microfluidic co-flow nanoprecipitation technique. Initially, the PLGA and therapeutic agent were added acetonitrile, and the outer fluid was lecithin with DSPE-PEG-2000 dissolved in a 4% ethanol–water solution. Both fluids were introduced into the microfluidic device using separate inlets at a different flow rate (1:5—1:50 mL/hour). When these two immiscible fluids were combined in a glass capillary, they precipitated and permitted the creation of self-assembled LPHNPs after 2 h of stirring at 800 rpm. The bulk nanoprecipitation approach was also employed to make LPHNPs for comparison. The LPHNPs developed by the bulk nanoprecipitation technique, the obtained data revealed that the formulations prepared by the microfluidic approach had good core–shell morphology, indicated relatively higher % EE, and controlled release of the SFN from LPHNPs. The SFN release from LPHNP formulations followed the Higuchi model with the Fickian diffusion mechanism. In vitro cell viability studies on MDA-MB-231 and PC3-MM2 cell lines revealed that the SFN-loaded LPHNPs prepared with a microfluidic approach suppressed cell growth more effectively than the SFN-loaded LPHNPs prepared with a bulk nanoprecipitation approach and free drug solution [181]. Epithelial to mesenchymal transition (EMT) is known for breast cancer metastasis and chemoresistance. Therefore, inhibition of EMT can result in cancer cell drug sensitivity and death. To achieve the same Zou and group (2021) developed an Emodin drug encapsulated LPHNPs by nanoprecipitation method (Fig. 7). The in vitro result suggested that the delivery of emodin via LPHNPs increased DOX and galunisertib sensitivity in drug-resistant MCF-7/ADR cells. The western blotting confirmed the down regulation of EMT markers (N-cadherin and vimentin) in drug-resistant cell line after treatment with emodin-loaded LPHNPs and galunisertib or DOX [182].

Going a step forward, LPHNPs with pH sensitivity was developed and assessed for breast tumor. The NPs were prepared by self-assembled nanoprecipitation technique encapsulating DTX. A higher release of DTX with approximately 42% drug release was observed within 12 h at acidic pH (5.5), while only 20% release of DTX was established after increasing the pH to 7.4. The time and concentration dependent reduction in MDA-MB-231 and MCF-7 cell viability was seen for pH sensitive drug loaded LPHNPs than non-pH sensitive drug loaded NPs and free drug. This higher toxicity attributed to endosomal escape of the NPs via aggregation of NPs in presence of acidic pH of the endosome. The in vivo study in Balb/c female mice showed enhanced target-specificity, lower tumor burden and pharmacokinetic with lower drug circulation in the deep-seated organs in the case of DTX loaded pH sensitive LPHNPs as compared to the non-pH sensitive LPHNPs-DTX and free DTX [183]. In the same lane, magnetic field responsive LPHNPs was reported in breast cancer drug delivery [61]. For this, LPHNPs were designed in way that the iron oxide and CPT can be encapsulated within the NPs. The hydrophobic polymer encapsulates the hydrophobic drug and releases the drug on demand under the influence of radio frequency (RF) magnetic field which causes loosening of polymeric cores by localized heating of Fe_3_O_4_. The iron oxide NPs embedded within lecithin and DSPE-PEG and PLGA was synthesized. CPT was encapsulated during the synthesis of the LPHNPs. Because of their stimuli-responsive features, LPHNPs displayed adjustable drug loading (from 1-10wt%). The magnetic field-assisted drug release was tested by applying a remote radio frequency of 100 kHz, and the drug release was found to be nearly 100% in 48 h. The viability of MT2 mouse breast cancer cells was investigated in vitro following treatment with different blank and drug-loaded LPHNPs. Due to regulated radio frequency, the CPT-loaded LPHNPs had a much lower relative MT2 cell growth rate than the non-stimulated formulation. The platform's specialties were simplicity of synthesis, stability qualities, and a regulated drug release approach, all of which might enhance cancer treatment [61].

Targeted LPHNPs have an added advantage in the cell-specific delivery of drugs [94, 194–198]. The cancer cell-specific overexpression of some receptors makes the uptake of the NPs easier. In this concern, cyclic RGD-modified LPHNPs were used to escort 10-hydroxycamptothecin (HCPT) to human breast tumor cells. Here, the hydrophobic drug HCPT was loaded inside the hydrophobic core of PLGA polymer, while lipid coating increased the stability and biocompatibility of the NPs. Further, surface functionalization with cRGD increased the effective targeting of LPHNPs to α_∨_β_3_-positive breast cancer cells. The LPHNPs were made using a slightly modified emulsification solvent evaporation process. These LPHNPs were incubated with bovine serum albumin (BSA) for 24 h to study the protein-LPHNPs interaction. The results revealed that 42.5 µg protein was adsorbed per mg LPHNPs. The drug release investigation revealed an early burst drug release pattern of 54–62%, linear drug release up to 3 days after 24 h, and then continuous release up to 10 days. After ten days, the overall drug release was 72–77%. The MDA-MB-435 s cells took more cRGD modified LPHNPs in cellular uptake assays, and this absorption was considerably greater than non-targeted LPHNPs. According to in vitro cytotoxicity experiments, the IC_50_ value of cRGD modified CPT-loaded LPHNPs was 0.262 µM against MDA-MB-435 s and 3.845 µM against MCF-7 cell lines. While the IC_50_ of free CPT was 0.723 µM against MDA-MB-435 s and 5.710 µM against MCF-7 cell lines. This study concluded that, compared to non-targeted and free drug solutions, the cRGD modified CPT-loaded LPHNPs were the most appropriate and promising tool for treating breast cancer [188]. A unique cRGD peptide which has the binding ability for α_v_β_3_ integrin receptors, α_v_β_5_ integrin receptors and Nrp1 receptors, was mounted over LPHNPs for targeted delivery of isoliquiritigenin (ISL). The targeted LPHNPs were synthesized by a modified one-step nanoprecipitation approach with soya lecithin, DSPE-PEG-2000 and PLGA. Drug-loaded iRGD modified LPHNPs formulation inhibited the growth of MCF-7, MDA-MB-231, and 4T1 cells better at 1.5625 µM ISL due to effective internalization of NPs inside the cancer cells. Similarly, cell lines treated for 16 h with drug-loaded iRGD modified LPHNPs had a 40% greater incidence of apoptosis than cells treated with conventional formulations. The in vivo anticancer activity was tested using 4T1 cells bearing nude mice models, which showed decreased tumor volume (474 mm^3^) and reduced mean tumor burden via drug-loaded iRGD modified LPHNPs. The investigations demonstrated that by employing drug-loaded iRGD modified LPHNPs for 4T1 cells, the dose required was lowered by almost half, from 50 mg/kg/day to 25 mg/kg every two days. The findings suggested that constructing iRGD modified LPHNPs would be a potential method for targeting breast cancer [189]. Table 1 summarizes various strategies for treating breast cancer via LPHNPs.

In this series, Zhang et al. (2015) used thin-film hydration and ultrasonic dispersion methods to accomplish folic acid (FA) modified lipid-shell and polymer-core NPs (F-LPHNPs) for the targeted distribution of PTX [199]. Here, the polymeric core of ε-caprolactone holds the PTX while FA on the lipid surface acts as a targeting moiety. The receptor-mediated endocytosis mechanism of the formulation was studied in EMT6 breast cancer cells overexpressing folate receptor overexpressed and L929 fibroblast cell lines (folate receptor-deficient). The F-LPHNPs fluorescence was brighter than regular LPHNPs in the cytoplasm of EMT6 cells when Nile-Red dye was utilized as a fluorescence indicator. The PTX-loaded LPHNPs and folate-modified PTX-loaded LPHNPs were used in an in vitro cytotoxicity study using EMT6 cells for a comparative study. Cytotoxicity of PTX solution was greater due to the direct availability of the drug than folate modified LPHNPs and bare LPHNPs. Although, the cytotoxicity of folate-modified LPHNPs was greater than normal LPHNPs. The results showed that killing tumor cells requires a larger drug concentration (25 µg/ml) and a longer incubation time (at least 72 h) due to sustained release of the drug from LPHNPs. They used EMT6 tumor-bearing BALB/c mice for in vivo anticancer investigation. According to the findings, folate-modified PTX-loaded LPHNPs showed a significantly higher circulation time, sustained release of the PTX, and higher inhibition of tumor growth (65.78%) than conventional PTX-loaded LPHNPs (48.38%) [199].

Cancer treatment is always compromised due to low aqueous solubility, strong toxic reactions affecting multiple organs, non-targeted delivery, cancer cell heterogeneity and drug degradation. Thus, as a delivery system, nano-carriers offer plethora of advantages [82, 200–207]. Taking about particular drug delivery nano-vehicle, they could be of two types: either polymer or lipidic nanoparticles. The lipoidal monomers, depending on the type of phospholipid, could bear varied structural identity, having striking pharmacokinetic profile, modifiable surface, excellent biocompatibility and high loading efficiency [208–213]. But, the most detrimental fact concerning them is low encapsulation, speedy release and instability upon long term storage. Moving towards polymeric nanoparticles, such limitation could be ameliorating with uniform release of drug, stability, acceptable size and importantly enhanced encapsulation efficiency. The idea of amalgamation, could possess a quality of one nanomaterial with the advantage of other, exhibiting a next level approach in drug delivery.

**Fig. 8:**
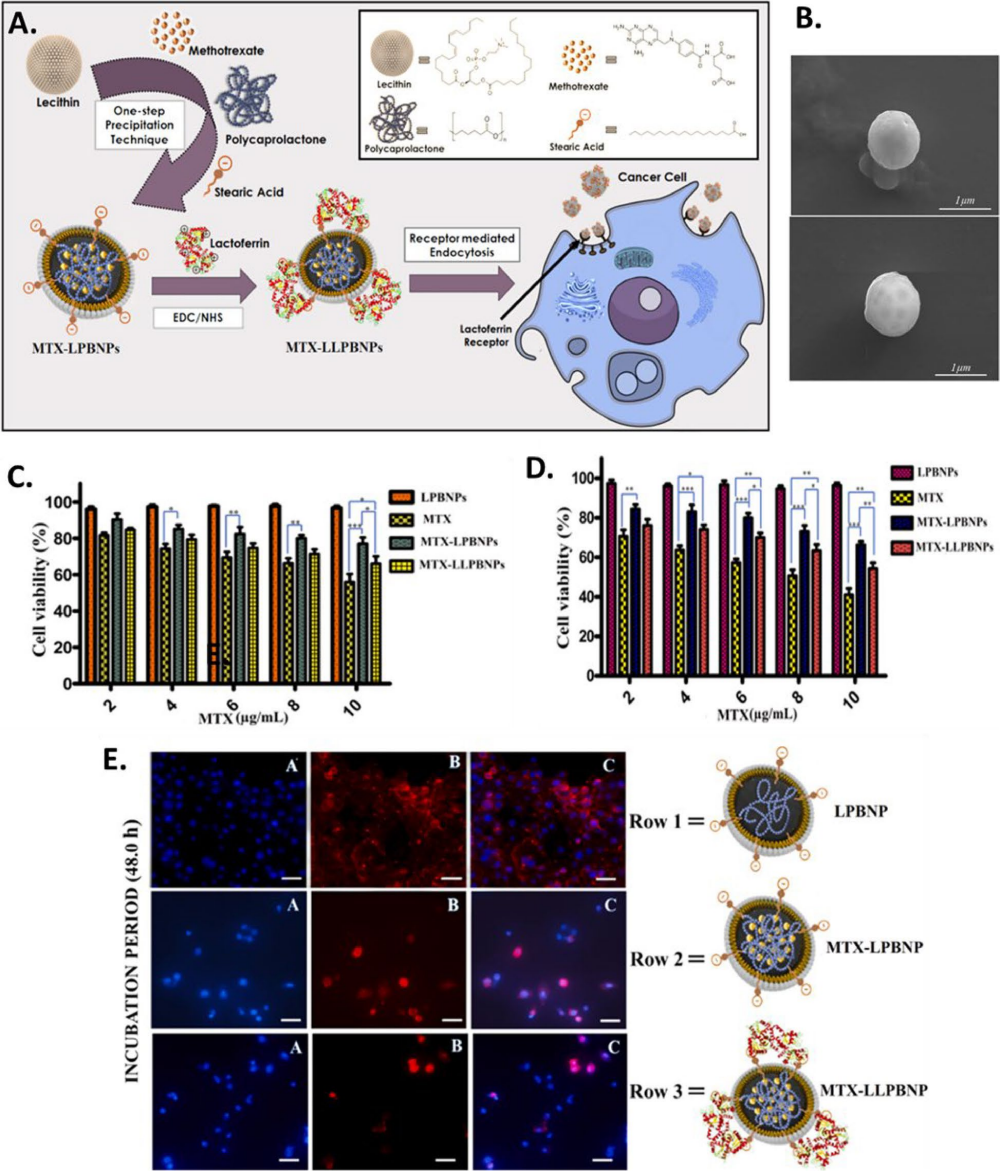
Representation of development of MTX loaded hybrid nanoparticles conjugated with lactoferrin as targeted delivery agent against cancer, (**A**) Illustration of development of targeted hybrid nanoconstructs using single step precipitation method along with its internalization following receptor mediated endocytosis, (**B**) Scanning electron microscopic images of lactoferrin un-conjugated (up) and conjugated (down) hybrid nanostructure, Cell viability study on MCF-7 cells treated with various preparations after (**C**) 24 h and (**D**) 48 h, (**E**) Representation of fluorescence microscopic study for ascertaining the uptake of lactoferrin conjugated and unconjugated nanoparticles. Reproduced with permission from Kumar et al. [214]. Copyright (2022) Elsevier

**Fig. 9:**
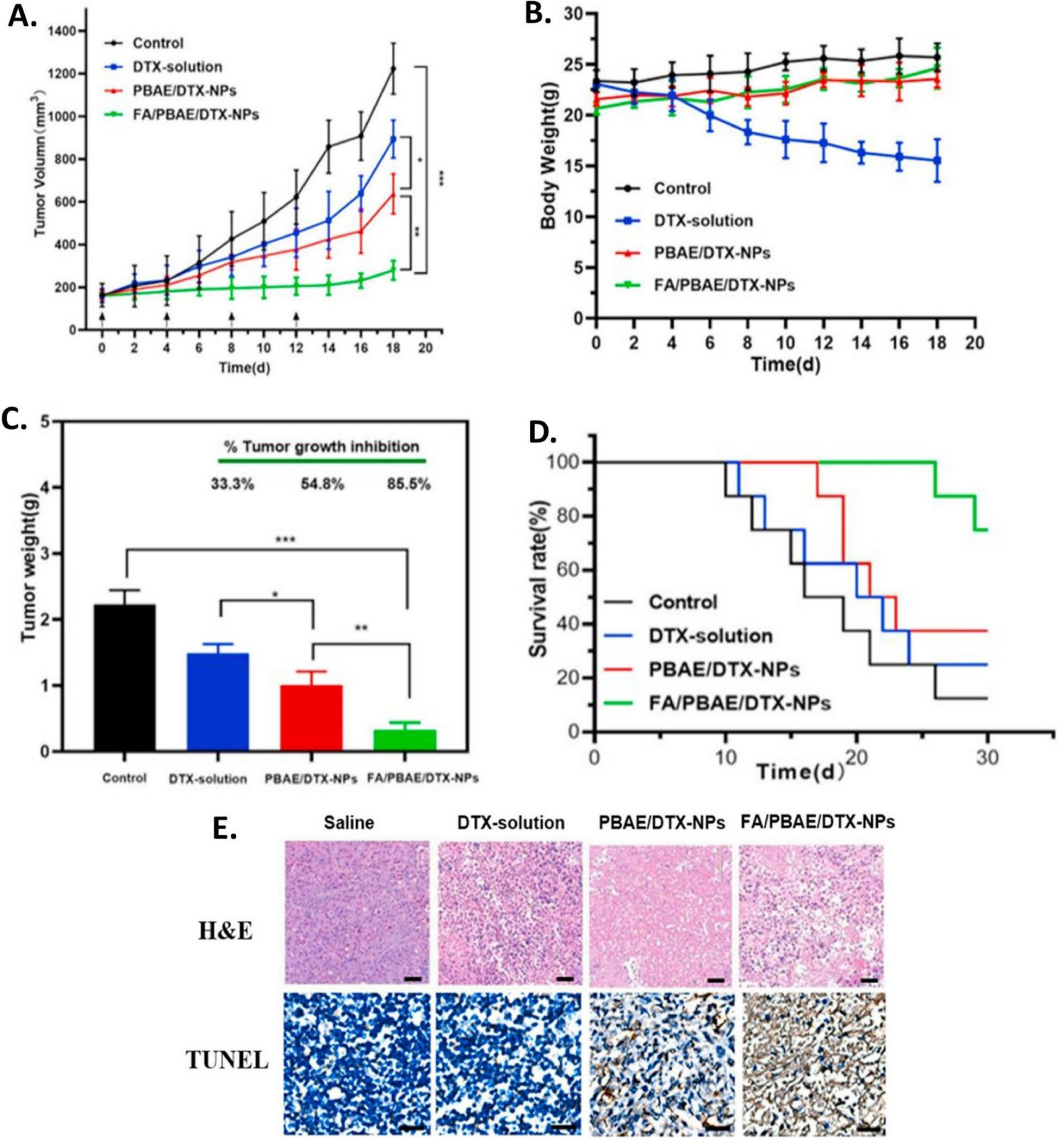
The anti-tumor effect of different formulation was evaluated in vivo, showing tumor growth (**A**), Weight variation of mice upon treatment (**B**), Tumor weight after sacrificing the mice (**C**), Percent survival rate (**D**), and microscopic images of tumor cells stained with H&E (**E**). Reproduced with permission from Zhang et al. [192]. Copyright (2022) Elsevier

Study offered by Kumar et al., based on same technique, developed a well-structured lipid-polymer hybrid nanoparticle involving methotrexate encapsulated in core made of polycaprolactone, while the shell comprised of stearic acid bearing soy lecithin. Through traditional cross-linking chemistry, the hybrid nanoparticles were made functional with lactoferrin. The developed nanoparticle has acceptable size range, potential and surface morphology. The ligand anchored nanocarrier showed excellent uptake by the cells with enhance cell reducing effect, exhibiting higher interaction of cell membrane with the nanocarriers (Fig. 8) [214]. Another study was conducted by Zhang et al. where they established pH responsive behavior of developed LPHNPs encapsulating docetaxel [192]. The cellular pH changes from one site to another, in a similar manner, the normal cells possess a physiological pH of 7.4, cancer cells extracellular matrix has a pH of 5 to 6.5 while for lysosome, it lies in between 4.5 to 5. Such a shift in pH could be useful to develop agents recognizing a drift in pH value, accumulate and release in required fashion. Poly (β-amino esters) or PBAE is one of such pH responsive polymer which is easy to synthesize and in accordance to change in environment breaks into small biodegradable compounds without causing toxicity in vivo. At normal physiological atmosphere, the PBAE huddles in the lipophilic region showing poor aqueous solubility, in contrast, after reduction in the pH, the solubility escalates due to the protonation of the amino group showing expansion of volume, thus escaping from lysosomal degradation. Thus, the researchers, developed PBAE in their laboratory through the Michael-addition reaction. Following the self-assembly and single emulsion method, the LPHNPs were developed using lipid (DSPE-PEG 2000). To achieve the targeting effect, the NP’s were modified with folic acid forming FA-PBAE-NP for escorting agents towards folate receptor over-expressing cancer cells. The shape of the nanoparticles remained constant at physiological pH, whereby, lowering of pH showed an increase in size which is due to the increment of hydrophilicity of the polymeric network in consequence of protonation of circumferential amino compounds. A strong cell killing effect with profound uptake the cancer cells was demonstrated which was comparatively more than the non-targeted nanoparticles, establishing higher internalization of targeted LPHNPs due to receptor-mediated endocytosis. Hence, to bring forth, LPHNPs by surface modifying potential could be advantageous in targeted cell death without affecting the normal cells (Fig. 9) [192].

#### Dual drug delivery

**Fig. 10:**
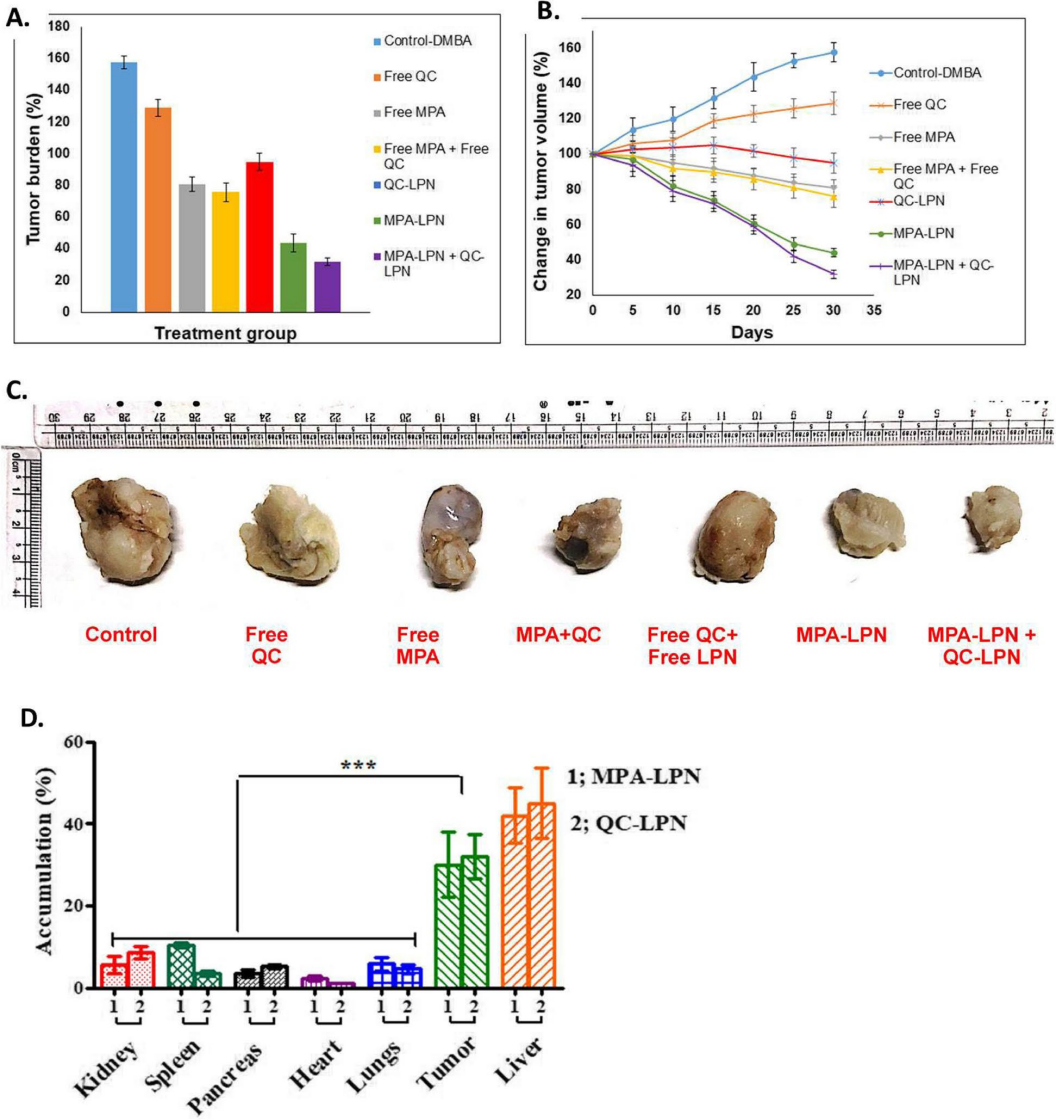
Representation of percentage change of tumor burden (**A**) and tumor volume (**B**) upon treatment with different preparations, (**C**) Excised grown tumor, and (**D**) The distribution volume of QC and MPA on distinct SD rats. Reproduced with permission from Patel et al. [152]. Copyright (2020) Elsevier

Due to the structural properties, it is easier to load more than one drug inside LPHNPs. Therefore, LPNHPs have been explored for simultaneous delivery of more than one drug. The lipophilic DOX and non-ionic compounds GG918 (Elacridar) co-loaded LPHNPs were created for managing the increased multidrug resistance in breast cancer treatment. It was the first approach to co-deliver P-gp inhibitor (GG918) and cytotoxic drug DOX via a lipid-based formulation to overcome drug resistance in breast cancer. The LPHNPs were made using an ultrasonic technique with minor modifications. In a biphasic fashion, the GG918-loaded LPHNPs demonstrated a slower release pattern than the DOX-LPHNPs. In the first 4 h, 50% of DOX and 25% of GG918 were released from LPHNPs. But, 72 h later, less than 40% GG918, and more than 60% DOX release were seen. The MDA-MB-435/LCC6/MDR1 cell lines were incubated with formulations to explore the cytotoxicity and chemosensitizing effects. After 24 h of incubation, cells exposed with DOX and G918 co-loaded LPHNPs had the lowest cellular integrity. Herein, GG918 increased DOX cellular absorption in p-gp-overexpressing cells. The clonogenic experiment also investigated how long-term DOX administration with and without GG918 suppressed cell growth. DOX and GG918 co-loaded LPHNPs had an IC_50_ of 0.34 mg/mL, which was much lower than the combination of free DOX and free GG918 (IC_50_ 0.94 mg/mL) [184]. In another study, the effect of PTX was enhanced by co-delivering it with miR-221/222 inhibitors which boosted the impact of calcium phosphate LPHNPs on triple-negative breast cancer therapy. The hydrophilic miRi-221/222 were encapsulated with calcium phosphate by co-precipitation method and the precipitates were then coated with an anionic lipid, dioleoylphosphatidic acid (DOPA), to co-encapsulate PTX. The release of miRi-221/222 from LPHNPs at different pH levels was found to be 40% at pH 5, and 20% at pH 7. This behavior was attributed to the dissolution of calcium phosphate at low pH conditions. The cytotoxicity investigation was conducted on the MDA-MB-231 cell line, and the findings revealed that cell viability was reduced up to 80% in the group that received the combination of drugs at a dosage of 0.67 µg/mL compared to the free or only PTX loaded LPHNPs (40%) at the same treatment concentration [185]. The LPHNPs have shown tremendous promise for the co-delivery of bioactives. The hydrophobic drug combination mycophenolate (MPA) and quercetin (QC) was co-administrated through LPHNPs in different structures for improved breast cancer therapy. LPHNPs were made using the one-step nanoprecipitation technique with pluronic F-68, soya lecithin, and DSPE-PEG. The sustained release of MPA (90%) was displayed for 48 h while QC release was slower as recorded in the release study. In vitro cellular uptake tests revealed that MCF-7 cells internalized C6 labeled LPHNPs more readily than free C6. In vitro cytotoxicity testing on MCF-7 cell lines revealed that the combined cytotoxicity of MPA and QC LPHNPs was higher than that of individual LPHNPs. The apoptosis indices of cells treated with QC and MPA LPHNPs, MPA-LPHNPs, QC-LPHNPs, free MPA and free QC were determined to be 0.91, 0.70, 0.44, 0.47 and 0.36, respectively. The in vivo antitumor efficacy of prepared LPHNPs was carried out using Sprague Dawley (SD) rats. A higher accumulation of MPA-LPHNPs and QC-LPHNPs was observed in tumor and liver tissues. The tumor size was determined to be 32.5% after 30 days of treatment with a combination of MPA and QC LPHNPs, compared to 154.59% in the control group (Fig. 10). The group getting LPHNPs combination therapy had a greater survival rate than the other formulation-treated groups. The results concluded that combination therapy could be very effective in treating breast cancer which can further be augmented using LPHNPs [145].

Loading of the dual drugs in NPs with cancer cell targeting ability has shown promising outcomes against cancer. A one-step sonication approach was used to create LPHNPs for targeted administration of indocyanine green and cisplatin (CPT) that were modified with FA. Here, fluorescent dye indocyanine green was used to acquire photothermal activity in LPHNPs. The photothermal efficiency investigation was also carried out on LPHNPs, free ICG, and water, with thermal changes observed using an infrared thermal imaging camera under laser irradiation. The highest temperature rise for ICG-loaded LPHNPs was obtained to be 54.6 °C, which was greater than the temperature rises for free ICG aqueous solution (51.3 °C). The in vitro drug release profile with and without laser irradiation was done at various time intervals, with the drug release without laser irradiation and with laser irradiation being 16.03% and 31.97%, respectively, at 12 h. The significantly enhanced early and late apoptosis of MCF-7 cells results were observed with the combined effect of LPHNPs plus laser (97.28%) in comparison to those only treated by laser (7.05%) or only LPHNPs (72.80%) [190]. Instead of using conventional ligands for targeting, researchers are trying to investigate diverse carbohydrates as effective cancer cells targeting moiety. For the same, fructose was tethered over LPHNPs for effective breast cancer therapy. The development of fructose-bounded LPHNPs was carried out that were loaded with methotrexate (MTX) and beta-carotene (BC) [Fu-BC-MTX-LPHNPs] as a breast cancer therapy. The MTX and BC co-loaded LPHNPs were made using a one-step nanoprecipitation process. The % scavenging activity investigation found that BC and MTX in combination and BC alone were equal, implying that only BC was responsible for scavenging activity, not MTX. In vitro cytotoxicity study was performed on MCF-7 cells. After 72 h of incubation, the viable cell counts of cells treated with F-BC-MTX-LPHNPs was less than 13% which was much less than BC-MTX-LPHNPs (21%), BC-MTX (24%), plain MTX (31%), and plain BC (65%). Fu-BC-MTX-LPHNPs showed a cellular apoptotic index of 0.87 against MCF-7 cells, more significant than the simple combination of BC-MTX (0.51). The in vivo anti-tumor activity in female Wistar rats displayed that the Fu-BC-MTX-LPHNPs treated group exhibited a residual tumor of 32%, which was significantly less than the combination of plain BC and MTX group (57.6%) and free BC treated group (84.7%). In contrast, the control group showed increased initial tumor volume to 147.3 ± 4.28% after 30 days. The study showed that co-administration of BC and MTX via fructose functionalized LPHNPs significantly reduced MTX-related toxicity and provided a synergistic anticancer effect for breast cancer treatment [186]. In this manner, fucose anchored LPHNPs were reported for co-delivery of MTX and aceclofenac (ACL) to treat breast cancer [79, 187]. The MCF-7 and MDA-MB-231 cell lines were used for cellular uptake studies. Fluorescence showed by cells incubated with fucose conjugated LPHNPs was much higher than free Coumarin-6 (fluorescence agent) and un-functionalized LPHNPs. The results indicated that fucose-conjugated LPHNPs entered the microenvironment of tumor cells very quickly through fucose receptor-mediated endocytosis. The cell viability assay results showed that a combination of ACL and MTX displayed more decreased cell viability than free MTX. After 72 h of incubation with fucose conjugated MTX-ACL-loaded LPHNPs at a dose of 20 µg/mL, the percentage of viable cell count was only 12–15%, which was lesser than other formulations. The surface expression of inflammatory mediators on MDA-MB-231 cells was assessed, and it was found that fucose conjugated MTX-ACL-loaded LPHNPs showed no surface expression of matrix metalloproteinase-1 (MMP-1). However, a marked increase in MMP-1 inflammatory mediators was observed on the surface of cells treated with other formulations. The in vivo anticancer activity was observed in the induced breast cancer model, which showed a reduction in tumor volume for groups treated with MTX-ACL combination (49.67%), significantly less than free MTX (73.66%). Similarly, the residual tumor burden for the group treated with fucose conjugated MTX-ACL co-encapsulated LPHNPs was found to be 19.54% which was significantly lower than MTX-ACL-loaded LPHNPs (33.73%), and normal saline (163.8%) treated group [187].

Because of their ability to self-renew and produce many lineages of offspring, cancer stem-like cells (CSCs), also known as tumor-initiating cells, have indeed been recognized and proposed to be a major contributor in therapeutic resistance and cancer recurrence [215, 216]. They have the ability to become dormant, transport drugs from the cells through overexpressed ATP-binding cassette transporters, actively repair DNA, scavenge reactive oxygen species (ROS), while being resilient to apoptosis [217, 218]. To this end, Shen et al., developed DOX and all-trans retinoic acid (ATRA) loaded into the outer membrane of lipid and inner surface of the polymeric system. Most tumors exhibit hypoxia as one of their distinguishing characteristics, and CSCs have been found to reside in the hypoxic niche, which helps to preserve their stemness. Once internalized by CSCs and accumulated in the tumor tissue, ATRA is quickly liberated due to the degradation of a synthetic lipid resulting in the liposomal shell's detachment by hypoxia dependent cleavage of the azobenzene link causing collapse of hybrid nanosystem. However, the release of DOX was comparatively slower due to the two spatial barriers-lipid bilayer and polymeric matrix. The anti-cancer study of the combinatorial preparation was determined on 4T1 cells. It was observed that 4T1 tumorsphere cells (TC) were resistant to DOX than the adherent cells. The cytotoxicity was markedly improved due to the synergistic effect having a combination index of 0.69. It is important to note that, pretreatment of 4T1 TC with ATRA improved the therapeutic index of dual drug loaded hybrid nanoparticle, reduced resistance, and elevated DOX sensitivity. The formulation also supported reduction of metastasis and inhibited tumor growth in vivo indicating promising role of dual drug therapy in cancer treatment [219]. Another study illustrated zein phosphatidyl choline hybrid nanoparticle for the treatment of aggressive triple negative breast cancer. The bioactive compound to act against tumor growth was isoliquiritigenin (ISL). The purpose of preparing the hybrid nanostructure was to enhance the drug loading efficiency, improve biocompatibility and improve industrial application. Overall, the preparation shown distinguished result both in vitro and in vivo [220].

#### Nucleic acid delivery

Cationic LPHNPs have been explored for systemic delivery of nucleic acids such as siRNA to treat breast cancer. Yang and group (2012) prepared cationic lipid ammonium bromide (N, N-bis(2-hydroxyethyl)-N-methyl-N-(2-cholesteryloxycarbonyl aminoethyl) and mPEG5k-PLA25k and PLA30k based LPHNPs. The positive surface groups of cationic lipids provided space for siRNA binding. The cytotoxicity investigation in human breast cancer cells BT747 revealed that blank LPHNPs at a concentration of 0.1 mg/mL had a cell viability of more than 90%, and that concentrations higher than this were not safe to utilize. In BT747 cells, the cellular absorption of produced siRNA-LPHNPs was examined. The FAM dye tagged siRNA was discovered within the BT747 cells after 1 h of incubation. The therapeutic target gene (Plk1) was also tested for downregulation by siRNA-loaded LPHNPs. After 24 h of incubation with siRNA-loaded LPHNPs carrying siPlk1 (200 nM) at N:P ratios of 5:1 and 10:1, gene expression was determined by qRT-PCR. The siRNA-LPHNPs had a higher rate of gene silencing (35.3%) than the control (100%). A comparative in vivo Plk1 gene silencing study demonstrated that siPlk1-LPHNPs after intravenous injection silenced the target gene by 65% as compared to PBS injected group [191]. In another study, cationic lipid dimethyl di-octadecyl-ammonium bromide-based LPHNPs were fabricated for IGF-1R (Insulin-like growth factor type I) siRNA delivery. In vitro cytotoxicity studies of lipid NPs and LPHNPs against MCF-7 cell lines were conducted. MCF cells treated with LPHNPs (300 µg/mL for 24 h) had a viable cell count of more than 90%, while cells incubated with lipid NPs at the same dose and period had a viable cell count of less than 20%. As a result of this finding, LPHNPs were shown to be more effective and safer to use. The cells incubated with siRNA-LPHNPs indicated a considerable down-regulation of IGF-1R expression in MCF-7 cells (*P* < 0.01) in comparison to control cells [221].

The appropriate planning of combinational use of multiple approaches to combat cancer could be a driving force in combating cancer with cancer cell plasticity and tumor heterogeneity. Till today’s date, the researchers are highly involved in treatment of cancer with the amalgamation of chemo-preventive and chemotherapeutic agents with the application of gene therapy. Earlier studies proved the importance and effectiveness of combinational anti-cancer approach by targeting certain key aspects involved in extension of malignancies [222, 223].

The cancer cells over express certain receptors which are actively involved in tumor progression and metastasis, challenging the developing world to come with better anti-cancer related ideas. The research, thus, now focusses on the targeted therapy to actively inhibit the growth of cancer lesions, reduce cell proliferation, growth and metastasis.

The insulin-like growth factor 1 receptor also called as Igf-1R, a transmembrane homodimeric receptor aggressively binds to its respective ligand known as insulin-like growth factor-1,2, activating the cell progression pathway involved in metastasis and suppression of apoptosis [221, 224–226] The incidence from history also proved its role in development of numerous cancers, including breast carcinoma. Such a theory confirms that targeting approach to delineate the effect of such receptors could be a meritorious tactic in cancer treatment [225, 227]. Using such technique, Mennati et al. for the suppression of Igf-1R, developed methoxypoly (ethylene glycol) and poly(caprolactone) nanoparticle for the delivery of lycopene and siRNA for targeting Igf-1R. Since, lycopene is poorly water soluble, its encapsulation by hybrid nanoparticle i.e., a shell of hydrophilic moiety and hydrophobic core seems to be applicable in drug delivery. The study demonstrated a well-developed spherical shaped structure with high entrapment efficiency of siRNA. Also, after the PCR study, the combinatorial treatment led to down regulation of Igf-1R, which was due to the effect of siRNA and lycopene. Cells treated with formulation without lycopene demonstrated cell arrest at S phase, however, the formulation with encapsulated lycopene showed cell cycle arrest in G1 phase. Thus, it could be established that lipid hybrid nanoparticles are suitable vehicle for dual delivery of lycopene and siRNA in the induction of cellular apoptosis [228].

### LPHNPs for the treatment of ovarian carcinoma

Ovarian carcinoma (OC) has the highest deaths among all gynaecological cancers. The ovarian surface epithelium (OSE) and surface epithelial inclusion cysts, which account for over 90% of original malignant ovarian tumors, are also known as epithelial carcinomas. Despite the large amount of research done in this field, progressive phases of OC are associated with substantial morbidity, mortality, and low survival rates. The low survival percentage of patients with OC is mostly due to a lack of potential early-stage identification screening technologies. Many strategies for treatment and control of OC are now accessible, including chemotherapy, radiation, combined cytoreductive surgery, combination chemotherapy, and debulking surgery. Recently, huge scope of nanotechnology put the light upon treatment of OC, which will be more effective in avoiding the negative side effects [229, 230]. Nanoparticles-mediated anti-cancer drug delivery can be used to target the OC. In this sequence, researchers have exploited LPHNPs as a carrier for delivering drugs to OC cells. Various approaches for the treatment of OC using LPHNPs have been summarized in Table 2.

**Table 2:** LPHNPs for effective ovarian cancer therapy

Sr. No	Lipid component	Polymer component	Targeting moiety	Drug	In vitro	In vivo	Ref
1	Egg phosphatidylcholine, DSPC and DSPE	PLGA	_	CPT	ES-2 human OC cells	_	[231]
2	Lipoid S75	Chitosan	_	CPT and CUR	A2780 cells	_	[232]
3	DSPE-PEG1k-NH_2_	PLGA	cRGD	Platinum IV	SKOV3	Female nude mice	[233]
4	Soybean lecithin, DSPE-PEG-2000-COOH, DSPE-PEG-2000-Folate and (1,2-dimyristoyl-sn-glycero3-phosphoethanolamine-diethylene-triamine-pentaacetate	PLGA	Folic acid	PTX and Yittrium-90	SKOV3	female Nu/Nu mice	[234]
5	DPPC, DPPG and DSPE-PEG (2000)-FA	PLGA	Folic acid	Indocyanine green and perfluoropentane	SKOV3	_	[235]

#### Single drug delivery

**Fig. 11:**
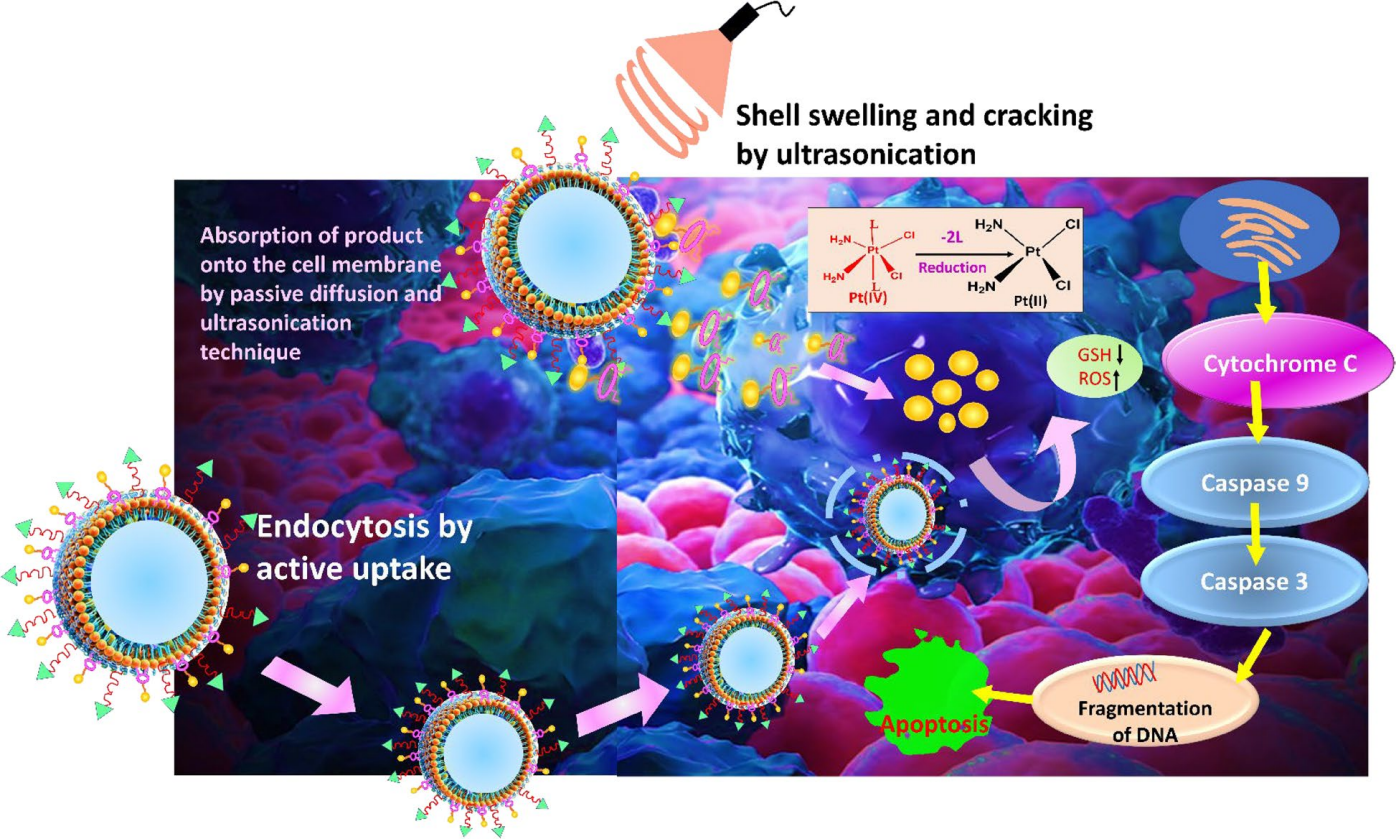
Mechanistic illustration of uptake mechanism of cRGD modified Pt (IV) LPHNPs via two distinct mechanisms (**a**) The prodrugs are actively up taken by passive diffusion and after sonoporation, particles interact favorably with the cell membrane leading to their absorption, (**b**) through interaction with integrins, particles are taken up via receptor mediated endocytosis

This category discusses studies wherein a single bioactive agent was delivered via LPHNPs to treat OC. The CPT-loaded LPHNPs were prepared with DOPC/DOPE–PEG-2000 and investigated for OC treatment. In vitro drug release of CPT from LPHNPs was originally discovered to be persistent and sluggish, fitting within a Higuchi order (*r*^2^ = 0.9801). The cytotoxicity of CPT-loaded LPHNPs in ES-2 human OC cells was tested in vitro. After 12 h of incubation, 60% of the ES-2 cells were viable and 30% after 24 h (*p* < 0.05) [231]. Another group of researchers developed GSH-sensitive Platinum IV (Pt IV) prodrug-decorated and cRGD functionalized LPHNPs for skilled theranostics against OC. The LPHNPs were made with a liquid core of perfluorohexane (PFH) and a mixture of PLGA12k-mPEG2k, PLGA12k-PEG2k-Mal, and DSPE-PEG1k-Pt(IV) as well as ligand. The drug release from LPHNPs after 24 h was determined to be 71.39 ± 5.20% with ultrasound at 20 mM GSH and 58.99 ± 5.33% without ultrasound at 20 mM GSH. The echo signal and contrast improvement were clearly visible in vitro and in vivo under ultrasound in the presence of targeted LPHNPs. The cellular uptake and in vitro cytotoxicity of cRGD modified LPHNPs were studied in SKOV3 human OC cells (α_v_β_3_- and α_v_β_5_-positive) and A2780 human OC cells (α_v_β_3_- and α_v_β_5_-negative). In SKOV3 cell lines, the cRGD alteration of LPHNPs boosted cellular uptake, while in A2780 cell lines, there was no significant improvement in cellular uptake. The viability of SKOV3 cells incubated with cRGD modified Pt (IV) LPHNPs at a dosage of 25 µM and ultrasound was determined to be 28.49%, whereas that of the same treatment without ultrasound was about 50%. The IC_50_ value of Pt (IV) LPHNPs modified with 1% cRGD against SKOV3 cells was obtained to be 20 µM. Similarly, the IC_50_ value of Pt (IV) LPHNPs without cRGD against SKOV3 cells was found to be 40 µM. In vivo tumor reduction studies of various formulations and free drug were carried out in SKOV3 tumor-bearing nude mice. A significant reduction in tumor volume was observed with ultrasound treatment for cRGD modified Pt (IV) LPHNPs compared to cRGD modified Pt (IV) LPHNPs without ultrasound and all other formulations (Fig. 11). The findings revealed that the cRGD modified Pt (IV) LPHNPs showed outstanding echogenic signals and synergies that increased the efficacy of the medicine for the treatment of OC [233].

#### Dual drug delivery

**Fig. 12:**
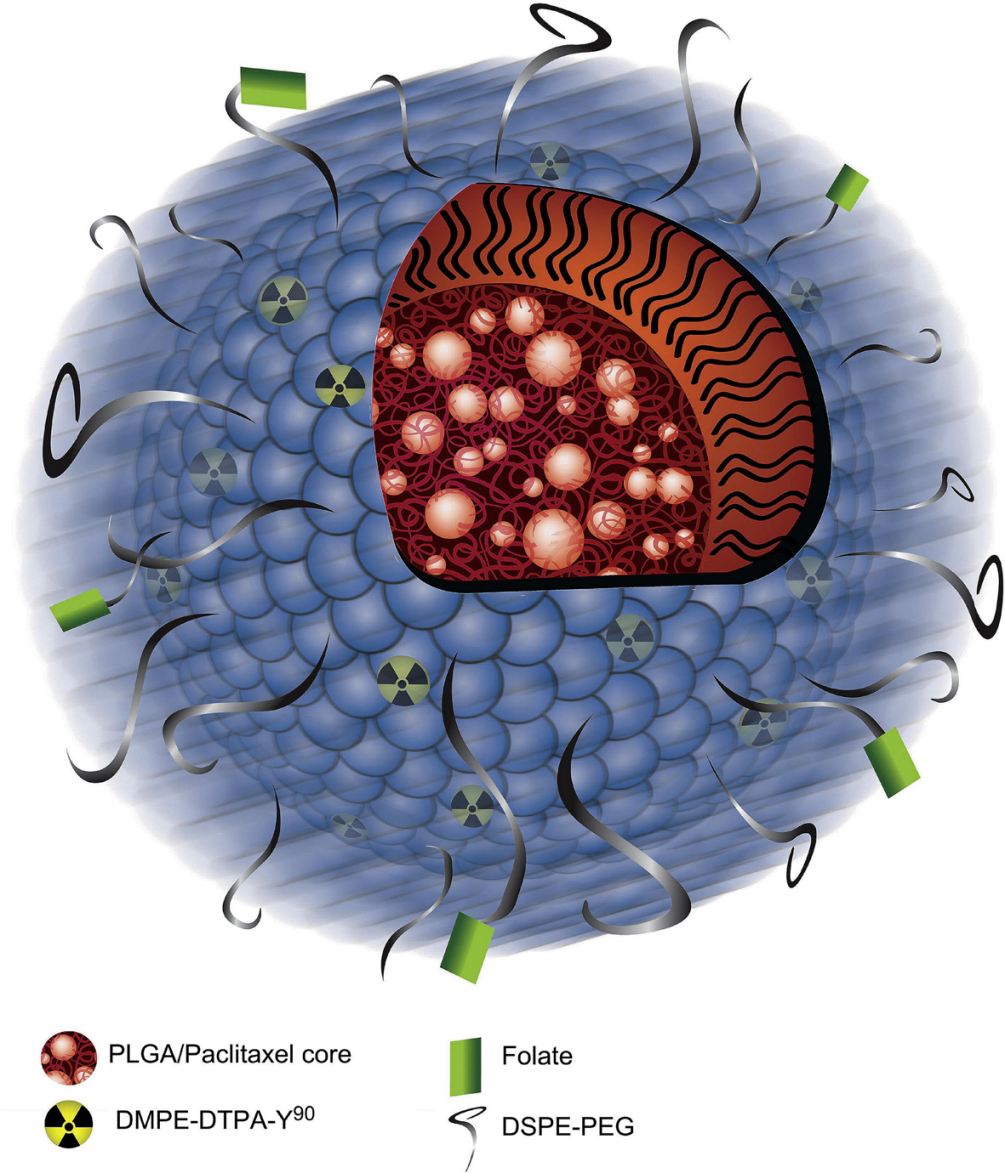
Depiction of the folate-targeted ChemoRad NPs. Reproduced with permission from [234]. Copyright (2011) Elsevier

A simultaneous dual drug delivery approach could overcome drug resistance in ovarian cancer cells. Khan et al. (2020) reported curcumin (CUR) and CPT-loaded LPHNPs to boost CPT cytotoxicity. They used the ionic gelation method to prepare the LPHNPs. The core was made of chitosan polymer, while lipoid S75 formed the shell. The EE and DL of CPT and CUR were varied over the lipid and polymer ratio. Both CPT and CUR were released in a controlled fashion from LPHNPs, while there was initial burst release in the case of free CPT and CUR. The release data displayed that, 50% of CUR and 68% of CPT were released in 24 h from LPHNPs. The cytotoxicity of synthesized LPHNPs was tested on A2780 cell lines. At a concentration of 6.2 and 3.1 µg/mL,

CPT and CUR co-loaded LPHNPs exhibited significantly enhance cell reduction. The results demonstrated that CPT and CUR co-loaded LPHNPs had much higher cytotoxicity. Similar results were obtained in a cytotoxicity study of various LPHNPs against a 3D spheroid tumor model. Compared to free CPT and CPT-LPHNPs, CPT and CUR co-loaded LPHNPs were more cytotoxic against 3D spheroid at 12.5 µg/mL concentration. This study demonstrated improved drug delivery for OC treatment using a co-delivered platform of nanocarriers [232]. For OC treatment, researchers used a self-assembly and nanoprecipitation approach to incorporate the PTX and radiotherapeutic agent (yittrium-90; ^90^Y) into LPHNPs. The LPHNPs were made more target-specific by introducing folate on their surface (Fig. 12). The folate receptor overexpressed SKOV3 and low folate receptor expressed SW626 OC cells were used in the in vitro cellular uptake investigation. The fluorescence of SKOV3 cells was much greater than that of SW626 cells. These findings demonstrated that the NPs were taken up via the folate receptor-mediated endocytosis pathway. The same cell lines were used to assess in vitro therapeutic efficacy. PTX and ^90^Y co-loaded LPHNPs and other formulations were incubated with the SKOV3, OVCAR3, and SE626 cells. The folate-targeted PTX and ^90^Y co-loaded LPHNPs were the most effective of all the formulations. SKOV3 cells treated with folate-targeted PTX and ^90^Y co-loaded LPHNPs had a survival fraction of 0.5, whereas cells treated with non-targeted PTX and ^90^Y co-loaded LPHNPs had a survival fraction of greater than 0.6. Similar findings were also seen in OVCAR3 cells. Furthermore, in vivo therapeutic efficacy was investigated in SKOV3 cells implanted female Nu/Nu mice. After developing the peritoneal metastasis of SKOV3 cells, mice were treated with 500 µg of different LPHNPs formulation. According to the findings, folate-targeted PTX and ^90^Y co-loaded LPHNPs outperformed non-targeted PTX and ^90^Y co-loaded LPHNPs and other formulations. The percent survival (after 50 days) in the folate-targeted PTX and ^90^Y co-loaded LPHNPs group was found to be over 80%, whereas in the non-targeted PTX and ^90^Y co-loaded LPHNPs group was found to be approximately 20%. More than half of the rats survived even after 90 days in the folate-targeted PTX and ^90^Y co-loaded LPHNPs group as compared to other treatment groups [234]. The folate-targeted LPHNPs loaded with indocyanine green (ICG) and perfluoropentane (PFP) were investigated for dual-directional characteristics. Here, ICG can act as a photodynamic therapy agent, while the PFP act as a contrasting agent for ultrasound imaging. The loading of both components inside LPHNPs was achieved via combining the two-step method and solvent evaporation technique. After 72 h, ICG release from LPHNPs was determined to be 25.95% in PBS and 29.05% in BSA, which was the lowest than other NPs formulations. The targeting impact of folate-targeted LPHNPs-loaded ICG/PFP and non-targeted ICG/PFP-loaded LPHNPs was compared in SKOV3 OC cells. Targeted LPHNPs have a considerably greater cellular absorption than non-targeted NPs. Targeted LPHNPs with photo sonodynamic treatment had a viable cell count of 16.39 ± 2.58% after 48 h, and the apoptotic rate was more than 80% [235].

### LPHNPs for the treatment of prostate cancer

Prostate cancer (PC) is one of the most aggressively developing and spreading disease in men, with a high death rate. Every year, millions of men throughout the world are impacted by this condition. Fusions of TMPRSS2 with ETS family genes, amplification of the MYC oncogene, deletion/mutation of PTEN or TP53 in late illness, and amplification/mutation of the androgen receptor are among the important genetic modifications in DNA sequences that cause this disorder (AR) [236–238]. To handle a high rate of mortality, general care or regulating techniques are insufficient. Surgery, radiation therapy, and chemotherapy are some of the common treatments. Apart from traditional medicines, other platforms such as drug delivery approaches, stimuli-triggered administration, hormone therapy, and most importantly, targeted therapy via nanomedicine are being explored to treat PC [239, 240].

Conferring to the application of computer-aided drug design, researchers are keen to optimize and develop nanoparticles with the help of design expert. On such note, Turk and team, worked on development of optimized core shell lipid-polymer nanoparticles loaded with piroxicam for treatment of prostate cancer. Overall, this cancer is difficult to treat with no significant treatment known so far. Considering this, the prepared core shell nanoparticulate structure showed a ray of hope due to the apoptosis potency and cytotoxic effect. The formulation resulted in crumbling of mitochondrial membrane potency while escalating the caspase level, demonstrating an improved effect in cancer therapy [241].

#### Single drug delivery

The delivery of therapeutic molecules via LPHNPs displays a promising strategy against PC. Zhang et al. (2008) used a one-step nanoprecipitation approach to make DTX-loaded LPHNPs for treating PC. The produced LPHNPs were stable in both 10% human BSA and human plasma solution. The DTX was released in a biphasic rhythm from LPHNPs. In the first 24 h, more than half of the DTX was released, and after that, a steady release was recorded for up to 120 h. The drug targeting efficiency was imparted by attaching amine-terminated A10 aptamer on LPHNPs, which can bind prostate-specific membrane antigen (PSMA). In PC3 PC cells, cellular uptake was investigated, and it was revealed that aptamer-anchored LPHNPs had a greater cellular uptake than non-targeted LPHNPs. The study demonstrated that aptamer-anchored DTX-loaded LPHNPs are a viable carrier system for PC therapy [242]. Similarly, CD44 antibodies coupled LPHNPs loaded with salinomycin (SM) were used for better targeting of PC initializing cells. The produced formulation demonstrated biphasic drug release, with early burst release (45% at 24 h) followed by sustained release (80% at 120 h). Fluorescence microscopy was used to investigate the capacity of LPHNPs to target cells in vitro by encapsulating PECF (a green fluorescent tracer). In CD44^+^ DU145 PC cells, the cellular absorption of PECF tagged SM LPHNPs coupled to CD44 antibodies was greater (mean fluorescence intensity was > 160) than cellular absorption in CD44^−^ DU145 cells (mean fluorescence intensity was 60). Similarly, in CD44^+^ 22RV1 PC cells, the cellular absorption of PECF tagged SM LPHNPs coupled to CD44 antibodies was greater (mean fluorescence intensity was > 180) than cellular absorption in CD44^−^ 22RV1 cells (mean fluorescence intensity was 80). In vitro cytotoxicity of SM-loaded LPHNPs coupled with CD44 antibodies was tested in PC cell lines DU145 and 22RV1. The IC_50_ values of SM-loaded LPHNPs coupled to CD44 antibodies against CD44^+^ DU145 cells and CD44^−^ DU145 cells were determined to be 1.4 ± 1.3 and 19.3 ± 6.8 µg/mL, respectively. Similarly, the IC_50_ values of SM-loaded LPHNPs associated with CD44 antibodies against CD44^+^ 22RV1 cells and CD44^−^ 22RV1 cells were 2.4 ± 1.6 and 20.8 ± 6.9 µg/mL, respectively. These results concluded that SM-LPHNPs-CD44 were selectively and effectively target CD44^+^ PC-initiating cells [243].

#### Dual drug delivery

Co-delivery of anti-cancer drug via NPs can have significant therapeutic efficiency against prostate cancer. DTX and CUR co-encapsulated LPHNPs were examined in vitro and in vivo for their increased anti-cancer efficacy against PC. The researchers developed LPHNPs using an amalgamation of self-assembly and nanoprecipitation technique. The MTT test was used to compare the cytotoxicity of free drug, DTX-CUR Co-loaded LPHNPs, non-lipid shell DTX-CUR loaded NPs, and single drug-loaded LPHNPs in PC3 cells. In comparison to other groups, results displayed the lowest cell viability for DTX-CUR co-loaded LPHNPs. The in vivo anticancer efficacy of LPHNPs was also assessed using a human PC-bearing Balb/c nude mouse model. Among the different treatment groups, DTX-CU co-encapsulated LPHNPs had the highest tumor inhibition rate of 82.5%, while DTX-CUR-NPs showed 62.1% inhibition of cells [244]. Similarly, Wang and group (2017) developed DTX-loaded core–shell type LPHNPs. These LPHNPs were co-loaded with an inhibitor of sphingosine kinase 1 (SK1) FTY720 (fingolimod) for the treatment of metastatic PC. LPHNPs exhibited pH-dependent drug release, which was quicker at pH 5 than at pH 7.4 and took 192 h to release the complete drug from the LPHNPs. The cellular internalization of Rhodamine B labeled LPHNPs was examined in PC3 and DU145 PC cells, displaying enhanced fluorescence intensity with increased incubation time. The effects of DTX and FTY720 on PC3 and DU145 cell lines were additive. The highest effective molar ratio of FTY720:DTX in PC-3 and DU145 cells was determined to be 5 M:5 nM, respectively. The dual drug-loaded LPHNPs containing the 5 µM:5 nM of FTY720/DTX showed cytotoxic effect of approximately 15%@24 h, 27%@48 h and 6%@72 h. The in vivo effect of LPHNPs was studied in NOD SCID gamma (NSG) immunodeficient nude mice xenografted with PC3 cells. The co-loaded LPHNPs (0.47 ± 0.06 g) and free drug (0.46 ± 0.07 g) significantly reduced tumor weight as compared to blank LPHNPs (g 0.75 ± 0.11 g) and saline (0.82 ± 0.12 g) treatment groups. However, the results showed that FTY720 (both free and in LPHNPs) allowed for a four-fold reduction in effective dosage and, more crucially, reduced FTY720-induced lymphopenia while suppressing other adverse effects, suggesting that it could be used in clinical PC treatment [245]. Prostate-specific membrane antigen (PSMA) targeted aptamer-functionalized, cabazitaxel (CTX) and CUR loaded LPHNPs (APT-CUR/CTX-LPHNPs) were developed for dual drug targeting in PC. Nanoprecipitation followed by self-assembly was used to synthesize desired LPHNPs. The investigators used LNCaP and PC3 cell lines to investigate cellular uptake. The targeted LPHNPs showed higher uptake by LNCaP cells as compared to non-targeted LPHNPs while low uptake was seen in PC3 by both the NPs. Herein, higher expression of PSMA on LNCaP cell lines was responsible for efficient cellular uptake of the targeted LPHNPs. The authors reported dose-dependent cytotoxicity of dual drug-loaded LPHNPs in PSMA positive LNCaP cell lines (*p* < 0.05). The pharmacokinetics and in vivo tissue distribution were investigated in xenografted BALB/c nude mice using an intravenous dosage of 2 mg/kg. The groups treated with APT-CUR-CTX-loaded LPHNPs showed better targeting and higher accumulation of drugs into the tumor. This unique combination of dual drug therapy showed high promise as a strategy for the effective treatment of PC [246]. Table 3 summarizes some of the LPHNPs formulations for treating PC.

**Table 3:** Application of LPHNPs for prostate cancer

Sr. No	Lipid component	Polymer component	Targeting moiety	Drug	In vitro	In vivo	Ref
1	Lecithin, DSPE-PEG,	PLGA	PSMA targeting A10 aptamer	DTX	PC3	_	[242]
2	DSPE-PEG-Malemide, PSC and cholesterol	PLGA	CD44 antibodies	Salinomycin	DU145 PC and 22RV1	_	[243]
3	Cholesterol and PSC and DSPE-PEG-2000,	PLGA	_	DTX and sphingosine kinase 1 (SK1) FTY720 (fingolimod)	PC3 and DU145	NOD SCID gamma (NSG) immunodeficient nude mice	[245]
4	Lecithin and DSPE-PEG	PLGA	_	CUR and DTX	PC3	BALB/c nude mice	[244]
5	SPC	PLGA-PEG-COOH	PSMA aptamer	CUR and CTX	LNCaP and PC3	BALB/c nude mice	[246]

### LPHNPs for the treatment of lung carcinoma

Lung cancer is a relatively common kind of malignant carcinoma that affects people all over the world (12.3 percent of all cancers), with a projected 1.2 million fresh cases detected each year. Small-cell lung carcinoma (SCLC) and non-small cell lung carcinoma (NSCLC) are the two most frequent types of lung cancer. The cells in both types of lung cancer begin to develop abnormally in distinct patterns and are treated differently. The incidence of NSCLC is higher than that of SCLC [247, 248]. In today's world, the rate of morbidity and death from lung cancer is alarming. The detection and treatment of lung cancer have piqued many researchers who want to try new ways. These unique techniques may be used to treat early-stage lung cancer and conduct several successful clinical studies. Different therapeutic methods have been explored, such as combination therapy through nanocarrier system, drug predilection, and targeted ligand conjugated drug therapy on relevant driver mutations [249–252]. There are a lot of reports on utilizing LPHNPs for the treatment of lung cancer (Table 4). This section discusses various strategies for lung cancer treatment using LPHNPs.

**Table 4:** Application of LPHNPs in lung cancer therapeutics

Sr. No	Lipid component	Polymer component	Targeting moiety	Drug	In vitro	In vivo	Ref
1	HSPC, DSPE-PEG-2000, DOTAP, DPPC and 1- palmitoyl-2-[6-[(7-nitro-2–1,3-benzoxadiazol-4-yl)amino]hexanoyl]-sn-glycero-3- phosphocholine	PCL	_	Erlotinib	A549 cells	_	[253]
2	Soybean lecithin SL-100 M and DSPE-PEG 2000	PLGA	_	HCPT	MCF-7	Kunming mice	[254]
3	DSPE-mPEG	amphiphilic polymer poly(ethylene glycol) methyl ether-grafted disulfide-poly(β-amino esters)	_	DOX	Lewis lung cancer cells	_	[255]
4	DSPE-mPEG_5000_ and soybean lecithin	PLGA	_	PTX and triptolide	A549	Balb/c-nude mice	[135]
5	3′-Dithiodipropionic acid,	PLGA	RGD	PTX and CPT	A549	Balb/c-nude mice	[256]
6	DSPE-PEG-2000-maleimide	PLA	EGFR ligand	CPT and DOX	A549	Male C57BL/6 mice	[257]
7	DOTAP	PLGA	_	Anti-inflammatory microRNA	Bronchial epithelial cells	_	[258]
8	DSPC-PEG-2000, Lecithin soybean and tristearin	PLGA	_	CD47 siRNA and etoposode	B16F10		[259]
9	Cholesterol-PEG	PLGA	Transferrin	Afatinib	H1975, PC-9	Balb/c-nude mice	[260]
10	CHO-PEG-NH2, SPC	Polycaprolactone (PCL)	Hyaluronic acid	Erlotinib and bevacizumab	A549, H1975 cells	Balb/c-nude mice	[261]

#### Single drug delivery

Erlotinib is an inhibitor of the epidermal growth factor receptor. The therapeutic efficiency of erlotinib was increased via LPHNPs delivery. Mandal et al. (2015) reported erlotinib-loaded core–shell LPHNPs (CSLPHNPs) for the treatment of NSCLC. The developed LPHNPs showed biphasic drug release and quickly released 50% erlotinib in 3 h followed by a sluggish release that lasted for 48 h. LPHNPs prepared with HSPC with NBD-PC (fluorescent phospholipid) were easily taken up by A549 cells*.* The in vitro cytotoxicity study in the same cell line revealed a dose-dependent cytotoxicity pattern. The IC_50_ of erlotinib-loaded LPHNPs was determined to be 100 nM after 72 h, whereas the IC_50_ of erlotinib solution was 2500 nM [253]. Further, for the delivery of hydroxycamptothecin (HCPT), LPHNPs synthesis was optimized with Quality-by-design (QbD) approach. The 2.50% HCPT was loaded inside the LPHNPs. The designed formulation greatly reduced IC_50_ values in MCF-7 (0.145 μg/mL) and HepG2 (0.220 μg/mL) cell line than free drug (0.494 μg/mL and 0.524 μg/mL) respectively, in same time duration (72 h). The HCPT-loaded NPs showed higher tumor regression with a single tail vein dose of 6 mg/kg in murine LLC-GFP-luc lung cancer-bearing mice, without severe side effects [254]. For highly controlled delivery of DOX, redox/pH-responsive LPHNPs have also been reported by Men and group (2019). LPHNPs were made from a self-assembled amphiphilic polymer poly (ethylene glycol) methyl ether-grafted disulfide-poly (β-amino esters) and PEGylated lipid. The pH-responsiveness of the synthesized LPHNPs was investigated through critical micellar concentration, which was found to be increased from 9.8 µg/mL to 37.1 µg/mL at pH 7.4 to 4, respectively. The destruction of LPHNPs was observed in transmission electron microscopy when the NPs were incubated with DL-dithiothreitol (reducing agent) for 4 h in PBS (pH 7.4) at room temperature. This confirmed redox responsiveness of the synthesized NPs. The same responsive phenomenon was observed in the drug release experiment, which showed < 30% and 90.1% DOX release from LPHNPs at pH 7.4 and 6.5, respectively, in 24 h. However, a more prominent DOX release (97.8) was observed at pH 6.5 with 10 mM DTT in the same period. The DOX-loaded LPHNPs were found to be more effective on Lewis lung neoplastic cells as compared to free drug and other formulations [255].

The ethnicity in association with expressing receptors are also the key factor for NSCLC metastasis and mutations. Likewise, epidermal growth factor receptor is also involved in accelerating the number of cancer cells, especially the polymorphism of EGFR.

The second generation tyrosine kinase-afatinib which is US-FDA approved drug is being used for treatment of cancer [262, 263].

Considering this, Wang et al. investigated the effectiveness of second-generation tyrosine kinase inhibitor-afatinib (US-FDA approved drug) entrapped in redox responsive, transferrin modified lipid polymer hybrid nanoparticles. This intended approach is due to the fact that in cancer cells, the level of cellular glutathione (GSH) is highly elevated, although, normal healthy cells do not exhibit such values. Firstly, the researchers developed transferrin (TF) modified redox sensitive ligand consisting of cholesterol and poly (ethylene glycol) forming ChOL-PEG-SS-TF and then was enveloped over drug containing LPHNPs. With increase in the concentration of GSH from 0 to 10 mM, drug from the nanoparticles tends to increase confronting the GSG dependent drug release. Such demonstrated behavior was due to presence of disulfide bond, which upon cleavage releases afatinib (AFT). The study conducted for developed nanoparticles demonstrated non obvious toxic result after treatment of cells with nanoparticles without drug, nevertheless, the treated cells with AFT loaded redox responsive targeted nanoparticles showed better cell inhibition effect, sustained release, higher blood circulation time, significant tumor inhibition in vivo and improved cell uptake [260].

#### Dual drug delivery

Dual delivery of therapeutically active agents could be more beneficial for lung cancer treatment. In this context, the PTX and triptolide (TL) dual-loaded LPHNPs were produced through nanoprecipitation. LPHNPs demonstrated sustained drug release, with about 90% of both drugs released in 48 h. The cytotoxicity of dual drug-loaded LPHNPs was dosage-dependent and effective against PTX-resistant A549 cells and non-drug-resistant A549 cells. The combination of PTX and TL with LPHNPs at 5:3 ratio was found to be the optimum. Incubation of PTX resistant A549 cells with PTX and TL co-loaded LPHNPs resulted in a PTX IC_50_ of 1.49 mg/mL and a TL IC_50_ of 0.89 mg/mL. PTX-resistant A549 cells xenografted nude BALB/c mice showed significant tumor regression (77.4%) as compared to the control group when injected with the dual drug-loaded LPHNPS. The findings showed that dual drug-loaded LPHNPs have higher therapeutic benefits and reduced systemic adverse effects [135]. In this sequence, RGD-tethered LPHNPs were reported by the emulsification-sonication approach to co-deliver PTX and CPT for lung cancer treatment. Both medicines were released in a biphasic manner by LPHNPs. The total drug release of both medicines in 16 h and 100 h was determined to be less than 30% and 80%, respectively. In A549 cells, cellular absorption of RGD modified LPHNPs was reported to be greater (> 60%) than that of basic LPHNPs (50%). The targeted LPHNPs aggressively entered into the A549 cells compared to human non-small cell lung cancer NCl-H1299 cells. The cytotoxicity experiment on A549 cells revealed that the IC_50_ values were 26.7 and 75.3 μg/mL for targeted PTX/CPT-loaded LPHNPs and PTX/CPT-free drugs combination. The results showed that the dual drug-loaded formulations had considerably higher anticancer activity than free drugs. Further, targeted PTX/CPT-loaded LPHNPs displayed significant tumor regression in A549 cell xenografted nude BALB/c mice compared to other formulations. The in vivo results confirmed that combinational targeted therapy via LPHNPs could be extremely beneficial for lung cancer treatment [256]. Likewise, LPHNPs loaded with CPT and DOX were applied for the treatment of lung cancer by targeting the epidermal growth factor receptor (EGFR). The solvent evaporation approach was used to make LPHNPs. The final LPHNPs had CPT in the hydrophobic polymeric core, DOX in the phospholipid layer, and an EGF-PEG-DSPE ligand layer on the outside. The DOX release was found to be easier than CPT from the LPHNPs and varied among the targeted and non-targeted NPs. The cytotoxicity studies in A549 cells exhibited that CPT and DOX in a 2:1 ratio had a synergistic effect with an IC_50_ of 0.57. In vivo studies demonstrated that the tumor inhibition rate in the group treated with EGFR-CPT/DOX LPHNPs formulation was over 74.5%, compared to roughly 20% in the group treated with a combination of free CPT and DOX. The findings revealed that EGFR-CPT/DOX LPHNPs were the most effective treatment for lung cancer [257].

**Fig. 13:**
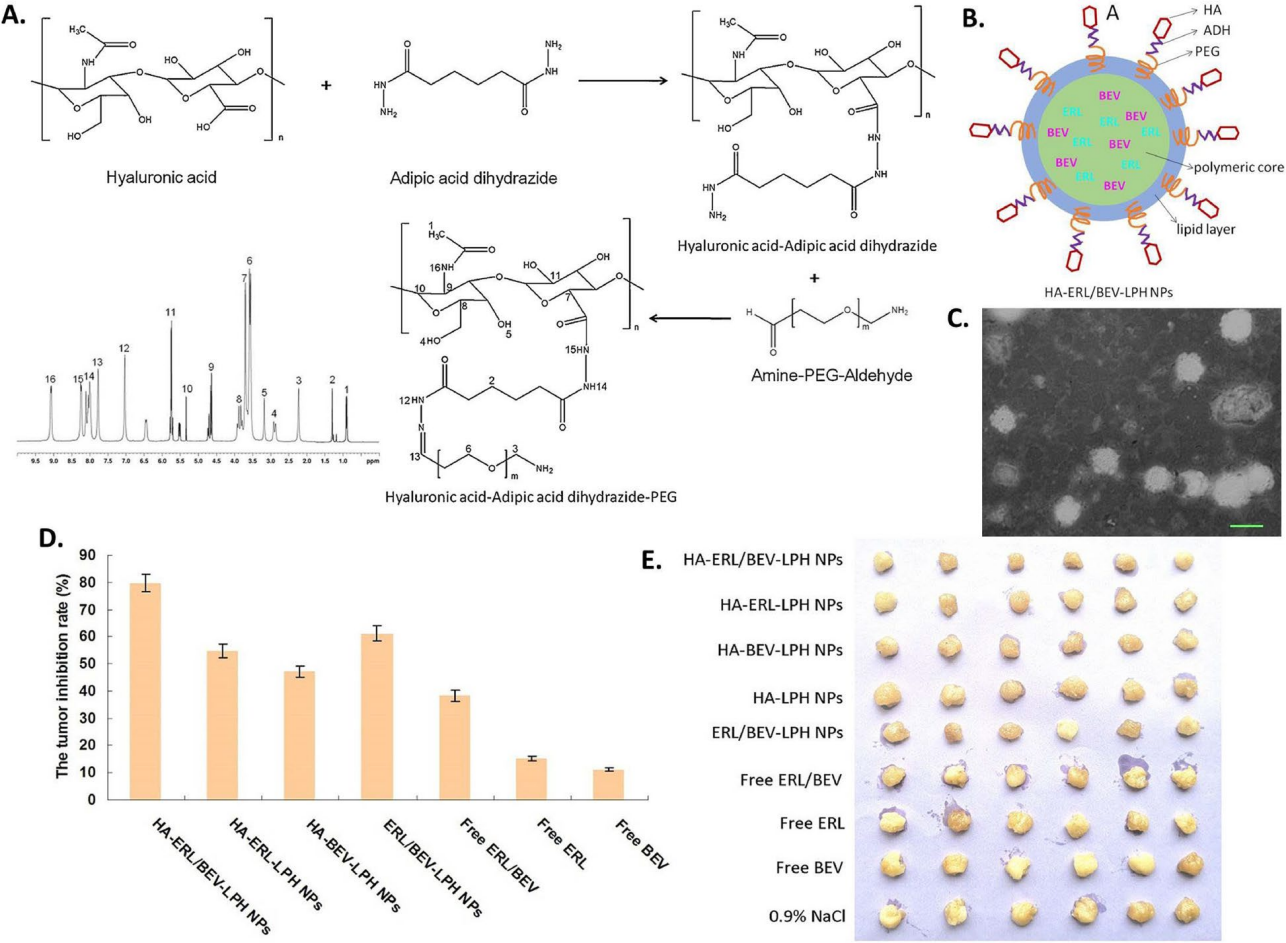
(**A**) Synthesis of hyaluronic acid-adipic acid dihydrazide linked with poly (ethylene glycol) caused by hydrazone linkage, (**B**) Schematic and (**C**) TEM image of hyaluronic acid linked erlotinib and bevacizumab loaded polymer lipid hybrid nanostructure, (**D**) Estimation of % tumor inhibition and tumor images after therapy, (**E**) Excised tumors. This figure is reproduced with Attribution-Non Commercial-No Derivatives 4.0 International (CC BY-NC-ND 4.0) license [261]. Copyright (2020) Elsevier

In cancer therapy, the key point to be considered is reduction of side effects and enhancement of therapeutic behavior. For mediating a target-based drug delivery and suppress the NSCLC, Pang et al. demonstrated the delivery of bevacizumab (monoclonal antibody against vascular endothelial growth factor receptor) and erlotinib (epidermal growth factor receptor inhibitor) via hyaluronic acid coated (CD-44 targeted) LPHNPs. The dual drugs loaded LPHNPs showed a synergistic anti-tumor behavior both in vitro and in vivo showing better results in comparison to single drug loaded nanoparticulate system. Additionally, the targeted NP showed long circulation behavior, better accumulation, pH sensitive drug release pattern with enhanced tumor inhibition assay. The particles revealed no deposition in cardiac and renal cells suggesting lower cardio and renal related toxic effects. Thus, a combination therapy of target based drug delivery could help to improve the survival rate of patients suffering from NSCLC (Fig. 13) [261].

#### Nucleic acid delivery

**Fig. 14:**
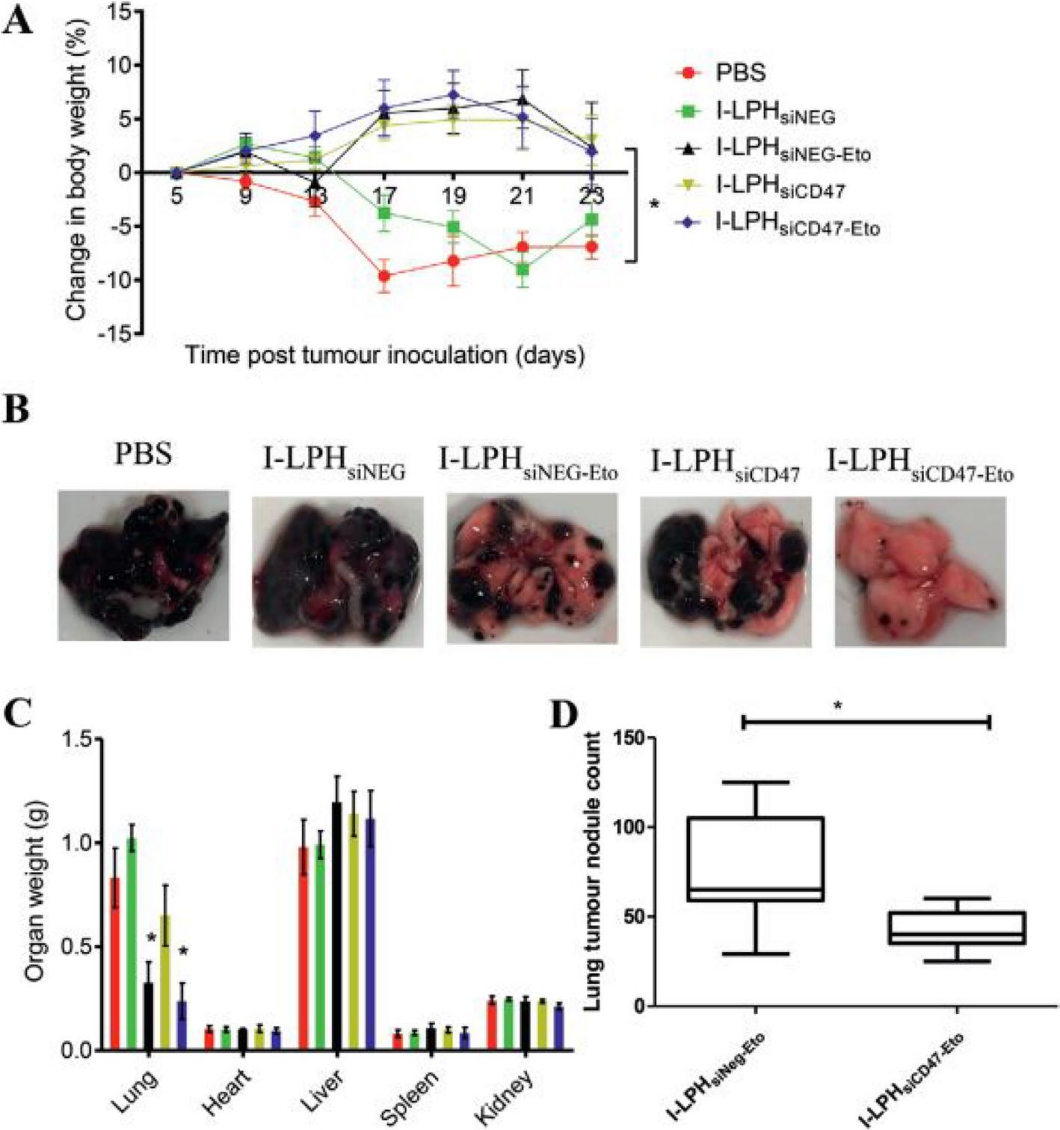
The dual drug loaded LPH preparation consisting of Eto and siCD47 showed better anti-cancer performance than the monotherapy post treatment. The treatment was provided on 2^nd^ and 7^th^ day with I-LPHsiNEG, PBS, I-LPHsiCD47, I-LPHsiNEG-Eto,, or I-LPHsiCD47-Eto. (**A**) Change in body weight (%) during experiment, (**B**) Representative lung images of different treatment groups, (**C**) Weight of the individual organ after sacrifice of the mice, (**D**) Lung tumor nodule count. This figure is reproduced under the terms of the Creative Commons CC BY license. Copyright (2021) John Wiley and Sons [259]

The major route of administration of LPHNPs has been intratumor or intravenous. However, the nasal route has also been explored for their delivery. Vencken and colleagues (2019) reported anti-inflammatory miRNA-loaded LPHNPs using an emulsion solvent evaporation process. These LPHNPs were nebulized for delivery of medicines to bronchial epithelial cells (BECs). LPHNPs were nebulized utilizing PLGA and cationic lipid 1,2-dioleoyloxy-3-(trimethylammonium) propane (DOTAP; a cationic lipid structure) in an Aerogen Solo vibrating mesh nebulizer. The cytotoxic effect of LPHNPs was investigated in NuLi-1 BECs cells. Apoptosis assay for Caspase-3 (an apoptosis marker) did not reveal any increased apoptotic impact in NuLi-1 BECs treated with 1 mg/mL LPHNPs compared to control culture but showed a slight increase in interleukin (IL) production. The miR-17-loaded LPHNPs, in comparison to negative control miRNA-loaded LPHNPs in high-density and low-density NuLi-1 BECs cultures, decreased the level of LPS-stimulated IL-8 secretion. This study found that DOTAP-modified PLGA LPHNPs were an efficient carrier method for miRNA delivery to BECs [258]. CD47 is a type of immunoglobulin which protect the cancer cells form mononuclear phagocytic system. Recently, co-delivery of CD47 siRNA and anticancer drug etoposode (ETO) were tried for lung cancer therapy. The stearyl amine was used in the preparation of LPHNPs for the effective loading of siRNA. siRNA was loaded either inside or outside of the prepared NPs. The B16F10 cell death assay showed 0.6611 and 0.1723 µM IC_50_ for free ETO and LPHNPs loaded with ETO, respectively. These LPHNPs demonstrated increased cellular absorption of siRNA in a concentration as well as the dosage-dependent manner and significantly silenced CD47 in B16F10 cells. In the in vivo biodistribution analysis, the formulation demonstrated a preferential uptake pattern into the lung, liver, and spleen. In an experimental pseudo-metastatic B16F10 lung tumor model, mice treated with dual treatment showed good therapeutic benefits (Fig. 14). Also, the ETO and siRNA-loaded LPHNPs inside the lung tissues revealed good immunological levels in CD4 + , CD8 + cells, and macrophages. The findings suggested that the combined chemo and immunotherapy could be better therapy for lung metastatic [259].

### LPHNPs for the treatment of hepatic carcinoma

Liver cancer or hepatic carcinoma is a serious cause of cirrhosis which is characterized by scarring and destruction to the upper right portion of the liver. Hepatocellular carcinoma (HCC) is the most prevalent kind of liver cancer that develops from liver tissue owing to aberrant hepatocyte and intrahepatic bile duct cell activity. Aggressive cell development also accelerates the demise of other related normal tissues and nearby organs [264, 265]. Various options for treating liver cancer with reduced systemic toxicity and minimal side effects are now available. Surgery, loco-regional treatment, dual or combination pharmacological therapy, liver transplantation, multi-kinase inhibitors, and immunotherapy are a few methods that can help with progression [266, 267]. The LPHNPs based hepatic carcinoma drug delivery was found to be a promising tool to cure liver cancer (Table 5).

**Table 5:** Application of LPHNPs for drug delivery to liver cancer

Sr. No	Lipid component	Polymer Component	Targeting moiety	Drug	In vitro	In vivo	Ref
1	Phospholipids and DSPE-PEG-2000	PLGA	_	Psoralen	HepG2/ADR	_	[268]
2	DSPE-PEG-Malemide	PLGA	EGFR antibodies	Adriamycin	SMMC-721	_	[269]
3	Soya lecithin, DOTAP	Hyaluronic acid	Hyaluronic acid	Sorafenib	HepG2	Kunming mice	[270]
4	DSPE-PEG-Malemide, Egg lecithin	PLGA	iRGD	DOX and Sorafenib	HepG2	Rat model	[271]

#### Single drug delivery

The benefits of LPHNPs have also been investigated for diverse drug delivery to liver cancer. Yuan et al., (2018) developed psoralen (PSO) loaded LPHNPs and investigated their reversal impact on cancer cell drug resistance. For the synthesis of LPHNPs, they employed the emulsification solvent evaporation technique. The in vitro release of drug from LPHNPs was shown to be biphasic, with a burst release of 30% in 2 h and a sustained release impact of 53% to 80% lasting up to 96 h. The in vitro cytotoxicity of PSO-LPHNPs was investigated in HepG2 cells after a 48 h incubation period. The IC_50_ value for DOX-resistant HepG2 cells was found 74.930 ± 0.82, 6.777 ± 0.46 and 3.254 ± 0.69 nmol/L after treatment with free DOX, PSO + DOX and DOX + PSO-LPHNPs, respectively. The results demonstrated that DOX and PSO-loaded LPHNPs were 23 times more effective than DOX solution in resistant HepG2 cells. The study found that dual-drug loaded LPHNPs had higher effectiveness against liver cancer [268]. In the subsequent research, a similar formulation (PSO-LPHNPs) was tested on P-gp over-expressing DOX-resistant cells [HepG2 (HepG2/ADR)] and investigated whether the formulation enhanced the efficiency of chemotherapy as compared to free PSO. In vitro, LPHNPs released 70% of their PSO in 24 h, followed by a very gradual release (> 90 percent) in 96 h. The in vitro therapeutic efficiency of formulation was investigated by exposing HepG2/S (drug sensitive) and HepG2/ADR (drug resistant) cell lines to PSO-LPHNPs and DOX for 24 h. The cytotoxicity of POS-LPHNPs was not observed against DOX-resistant HepG2 cell lines at concentrations less than 40 µM. DOX showed an IC_50_ value of 0.31 ± 0.06 µM against HepG2/S cells and 61.7 ± 9.3 µM against HepG2/ADR cells. In HepG2/ADR cells, co-administration of PSO-LPHNPs (PSO concentration 20 µM) and DOX resulted in a 17-times cytotoxic index compared to free DOX or DOX + PSO. After 24 and 48 h of incubation, the DOX + PSO-LPHNPs induced greater apoptosis in HepG2/ADR cells than free DOX or DOX + PSO treatment. However, none of these treatment groups modified the expression of P-gp in the hepatic cells [268].

In another study, cabazitaxel was enveloped in poly (lactic-co-glycolic acid) (PLGA) (as biopolymer for oral drug delivery) nanoparticle modified with poly (methyl vinyl ether-co-maleic anhydride) (PVMMA) (preferred as copolymer exhibiting strong gastrointestinal adhesiveness) and glyceryl monostearate to develop a hybrid nanoparticle. To inhibit the P-gp efflux, the nanoparticles were modified with D-α-Tocopheryl polyethylene glycol 1000 succinate (TPGS), thus developing TGPS-PLGA hybrid nanostructure (PTnp). Due to the addition of PVMMA and TGPS, the PTnp showed a slow release in the acidic medium which is due to the decline in hydrolysis of polyanhydride polymer in acidic pH, however, the pattern was not different at physiological pH, which is considered effective for intestinal absorption. Moreover, the PTnp has proved its oral retention property by intestinal retention and permeability test. PLGA, although had same zeta potential and size, had lower bioadhesive effect, suggesting the competence of PVMMA and TGPS in oral drug delivery. On similar note, PTnp, because of adhesiveness retained in middle portion of small intestine, existing mostly in duodenum and jejunum while, most PLGA nanoparticles were observed in the ileum portion. Not limiting to this, PTnp also reached the inner portion of villi due to the strong interaction between PTnp and mucin. Thus, the lipid hybrid nanoparticles opens the way of oral delivery of anti-cancer agents [272].

Nanocarrier systems have been explored extensively for targeted drug therapy for liver cancer and LPHNPs are one of them. LPHNPs loaded with adriamycin (ADR) and conjugated with anti-EGF receptor antibodies were prepared for the treatment of HCC. The LPHNPs-EGFRs were synthesized using one-step nanoprecipitation followed by a self-assembly approach. PLGA and soya lecithin were employed to make the polymeric and lipid phases. The controlled release was reported to be around 40% in the first 12 h, and then a very gradual release was detected, with just 65% after 24 h. The SMMC-7721, HepG2, and Huh 7 hepatocellular carcinoma cell lines were used in the in vitro cellular uptake study. Compared to LPHNPs of formulation and free ADR, the ADR-loaded LPHNPs-EGFRs demonstrated higher transfection efficiency and anti-tumor efficacy in SMMC-7721, HepG2 Huh 7 cells due to overexpression of EGFR. The in vitro cell line tests revealed that the IC_50_ for LPHNPs-EGFRs was 0.587 µg/mL and 1.299 µg/mL for LPHNPs alone in SMMC-721 cells [269]. Hyaluronic acid (HA) has been reported as a ligand for targeting sorafenib (SOR)-loaded LPHNPs to the HCC. The drug release from HA/SOR-LPHNPs was only 5.13%, but it accelerated significantly in the presence of HA enzyme, reaching 33.64% after 72 h. The flow cytometric results in HepG2 cells showed that LPHNPs were effectively internalized by CD44-mediated endocytosis. SOR-LPHNPs were shown to be cytotoxic to HepG2 cells in a dose-dependent manner. SOR-LPHNPs and free SOR solution had IC_50_ values of 2.73 ± 0.44 µg/mL and 8.84 ± 0.49 µg/mL, respectively. The lethal impact of SOR-LPHNPs was greatly decreased by pretreatment of HepG2 cells with free HA for 1 h, with an IC_50_ value of 4.19 ± 0.61 µg/mL, likely due to free HA molecules attaching to CD44. The in vivo targeting of produced LPHNPs was investigated in H22 tumor-bearing Kunming mice. Compared to the other groups, the group treated with SOR-LPHNPs had a good accumulation in the tumor (1.64 folds) and the most significant tumor growth inhibition (*P* < 0.01). This study indicated that HA conjugated SF-LPHNPs could be a promising technique to boost SF's anticancer effectiveness [270].

#### Dual drug delivery

**Fig. 15:**
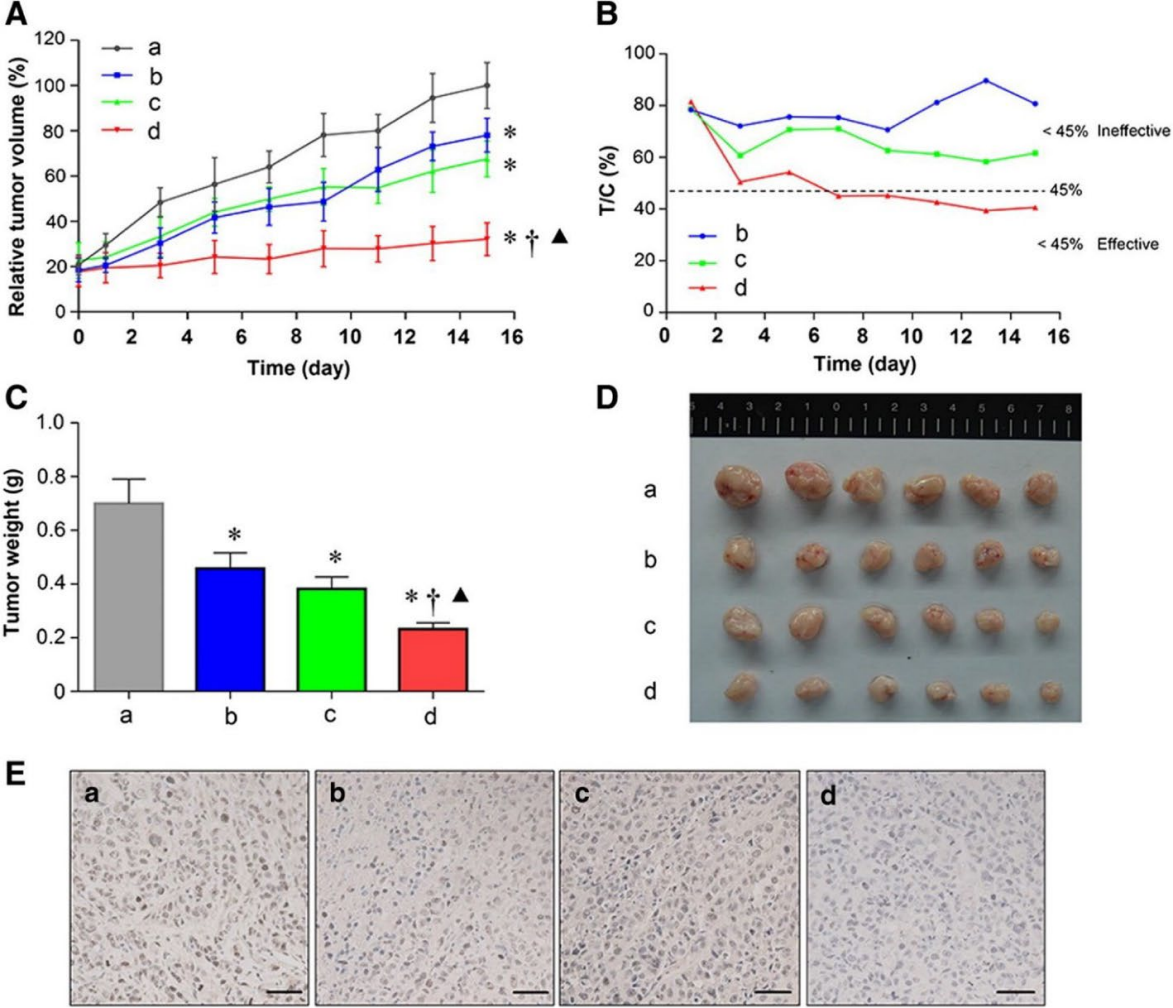
The in vivo anticancer effects evaluation on xenograft tumor model implanted with HepG2 cells. (**A**) Relative tumor volume in distinct groups, (**B**) Relative tumor growth inhibition rate, (**C**) Tumor weights in various groups, (**D**) Representation of excised tumor images after treatment with different formulations, (**E**) Immunohistochemical staining of Ki-67 expression in various groups. Reproduced with permission from Zhang et al. [271]. Copyright (2016) Elsevier

Targeted dual drug delivery via LPHNPs could be of great importance for the management of liver cancer. A modified nanoprecipitation approach was used to make DOX and SOR co-loaded iRGD decorated LPHNPs to improve anti-cancer efficacy in HCC treatment. The release of DOX and SOR from LPHNPs was 30% in the first 12 h and 80% in the next 144 h, indicating a gradual and persistent biphasic drug release pattern. For in vitro cellular uptake study, α_v_β_3_ positive HepG2 cells and α_v_β_3_ negative normal human liver L02 cells were used. When iRGD decorated LPHNPs were compared to regular LPHNPs, their cellular absorption was 2.5 times higher by HepG2 cells. In L02 cells, the iRGD alteration of LPHNPs had no significant effect on cellular absorption. The in vitro cytotoxic effect of prepared LPHNPs was studied in HepG2 cells. The HepG2 cells incubated with iRGD decorated DOX and SOR-loaded LPHNPs for 48 h showed IC_50_ values of about 0.3836 and 0.0765 µM for DOX and SOR, respectively. Similarly, HepG2 cells cultured over 48 h with a mixture of free DOX and free SOR revealed that DOX and SOR had IC_50_ values of 0.7631 and 0.1526 µM, respectively. The anticancer efficacy of produced LPHNPs was investigated in vivo in a HepG2 tumor xenografted rat model. The average tumor volume of the group treated with iRGD decorated DOX and SOR LPHNPs, for the group treated with free medicines suspension and for the group treated with both drug-loaded normal LPHNPs were 32.11, 78.07 and 67.53%, respectively, analysed in comparison to control group (Fig. 15). The designed formulation induced apoptosis markers like Cl-PARP, BAX and CI-Caspase 3 in tumor cells which were confirmed via western blotting. Overall, the iRGD decorated DOX and SOR co-loaded LPHNPs demonstrated improved antitumor efficacy in HCC xenograft mice models, implying a co-delivery strategy for HCC treatment [271].

### LPHNPs for the treatment of melanoma

Melanoma has been documented since ancient times. However, the relative incidence rates of melanoma have undoubtedly increased in the contemporary age as a result of modern sun-seeking behaviors [273]. The mutations in CDKN2A, mitogen-activated protein kinase cascade in sporadic melanomas, BRAF and NRAS, KIT, GNAQ and GNA11 genes and so on are basic reasons for melanoma in families [274]. Continuous radiation treatment and surgery are used in melanoma therapy to cure localized illnesses, but they have a number of dangerous side effects that can reduce one's quality of life. Newer techniques are meant to treat malignant conditions locally and systemically, increase drug reachability and acceptance to the desired location [275]. Table 6 summarizes LPHNPs based therapeutics in melanoma.

**Table 6:** Application of LPHNPs for the treatment of melanoma

Sr. No	Lipid component	Polymer component	Targeting moiety	Drug	In vitro	In vivo	Ref
1	DSPE-PEG, HSPC	PLGA	_	DOX	M14 melanoma cells	_	[276]
2	HSPC, Cholesterol, DSPE-PEG-2000	PLGA	Vitamin D	Fluorescein	B16	_	[277]
3	Lecithin, cholesterol	mPEG-PLGA	RGD	CUR	B16	female BALB/c mice	[278]
4	Hydrogenated phosphatidylcholine, DSPE-PEG2K-Maleimide	PLGA	Transferrin	Plumbagin	B16F10	female BALB/c mice	[279]

#### Single drug delivery

A melanoma cell bears vitamin D receptors on its surfaces and for targeting the same, Scopel and group (2020) synthesized vitamin D_3_ functionalized fluorescein LPHNPs by mild film hydration. The drug release from vitamin D_3_ functionalized fluorescein LPHNPs was ascertained in PBS (pH 7.4). In 24 h, there was a burst release of 57%, followed by a relatively gradual release of just 68% in 144 h. The in vitro cellular uptake demonstrated that the vitamin D_3_ functionalized LPHNPs were found in the cytoplasm of B16 cells after 3 h of incubation, while a longer time was required for the other formulation. These findings suggested that the vitamin D_3_ functionalized LPHNPs were well suited for drug targeted melanoma therapy. Targeted delivery via LPHNPs could be one such approach for melanoma treatment. Zhao et al. (2014) developed RGD functionalized CUR-loaded LPHNPs for cancer-targeted delivery and assessed them both in vitro and in vivo. The RGD modified LPHNPs were made with PLGA, m-PEG, RGD-PEG-cholesterol copolymers, and lipids. The safety of designed formulation was tested on HEK293 cells which showed 91% cell viability at 500 µg/mL of blank LPHNPs. The cell viability experiments on B16 melanoma cells revealed that the cell killing ability increased with the concentration. Still, there was no significant difference between the groups treated with free CUR and those treated with CUR-loaded LPHNPs. The antitumor impact of CUR-loaded LPHNPs was also investigated on the B16 tumor model (female BALB/c mice). After 9 days of therapy, the group treated with CUR-loaded LPHNPs demonstrated a substantial reduction in tumor development compared to other formulations. They also used the TUNEL immunofluorescence staining technique to evaluate tumor cell death. The apoptotic index in CUR-loaded LPHNPs group was 19.55% ± 2.51%, which was found higher than that in the free CUR (13.30% ± 3.05%, *p* < 0.001) and normal saline (2.57% ± 1.06%, *p* < 0.001) groups. Thus, the prepared LPHNPs showed improved results for the treatment of melanoma [278]. Recently, transferrin (TF) conjugated plumbagin (PL) entrapped LPHNPs were developed for melanoma regression. The in vitro drug release of produced LPHNPs was investigated at pH 7.4 and 5.5. Over the course of 24 h, LPHNPs released 81.7 ± 1.4% of their drug at pH 7.4 and 95.4 ± 0.7% at pH 5.5. In B16F10 cells, PL-absorption was shown to be 1.6-fold and 2.1-fold greater with TF conjugated LPHNPs than in cells treated with control LPHNPs and drug solution, respectively. The cellular absorption of TF attached LPHNPs was considerably reduced when all cells were pre-treated with 50 µM free TF. Further, the anti-proliferative activity of produced LPHNPs was tested in B16F10 cells which showed 3.2-fold extended cell killing activity by targeted PL-LPHNPs compared to other groups. TF conjugated PL-LPHNPs, control LPHNPs, and plumbagin solution had IC_50_ values of 0.16 ± 0.02, 0.31 ± 0.01, and 0.51 ± 0.02 µg/mL, respectively. B16F10 cells treated with TF attached PL-LPHNPs were found to be 89.2 ± 0.4% apoptotic, whereas those treated with control LPHNPs and PL solution were found to be 80.5 ± 0.6% and 27.5 ± 1.0% apoptotic, respectively. The in vivo tumoricidal efficacy of produced LPHNPs was investigated in B16F10-luc-G5 cancer cell-generated tumors (female BALB/c mice). The 40% of tumors from the TF-attached PL-LPHNPs group resulted in tumors vanishing, while 10% showed a partial regression and 20% were stable. The results concluded that TF-attached PL-LPHNPs could be a highly promising anti-cancer therapy for melanoma [279].

#### Dual drug delivery

To overcome DOX resistance, LPHNPs with a photo-releasing nitric oxide photodonor was created for treating melanoma. They used a modified two-step approach to make the LPHNPs. The photodonor loadings of nitric oxide and DOX were 0.85% and 0.68%, respectively. The visible blue light-dependent release of nitrous oxide was observed from DOX/NOPD-LPHNPs, which halted in the dark condition. They used DOX-resistant human M14 melanoma cells to test the biological activity of produced LPHNPs. The formulation triggers nitration of drug-resistant developing factor P-gp, MRP1, and BCRP when irradiated with blue light for 30 min. The inclusive outcomes exhibited a prominent augmentation in M14 cells killed by dual-action therapeutic LPHNPs than other formulations and free DOX [276].

### LPHNPs for leukemia treatment

Leukemia is a malignancy of the blood-forming cells, with a wide range of treatment options. The mainstay treatment for the most aggressive form of leukemias are chemotherapy in association with stem-cell transplant and radiation therapy. Nanotechnology has given rise to new methods for diagnosing and treating different leukemias that are easy and non-invasive. Smarter LPHNPs-based approaches have been explored to abolish these cancer cells with improved efficacy and enhanced specificity (Table 7) [280, 281].

**Table 7:** Application of LPHNPs for leukemia treatment

Sr. No	Lipid component	Polymer component	Targeting moiety	Drug	In vitro	In vivo	Ref
1	Cholesterol, oleic acid	Compritol 888 ATO	Transferrin	PTX	HL-60	_	[282]
2	DSPE-PEG	PCL	Hyaluronic acid	DOX, and gallic acid	Human K562 chronic myeloid leukemia	AML bearing mice	[283]
3	DPPC, DSPE-PEG-2000	PLGA	Engineered antibody	Tin mesoporphyrin	CD11b + myeloid cells	NOD-SCID il2r gamma − / − (NSG) mice	[284]

#### Single drug delivery

**Fig. 16:**
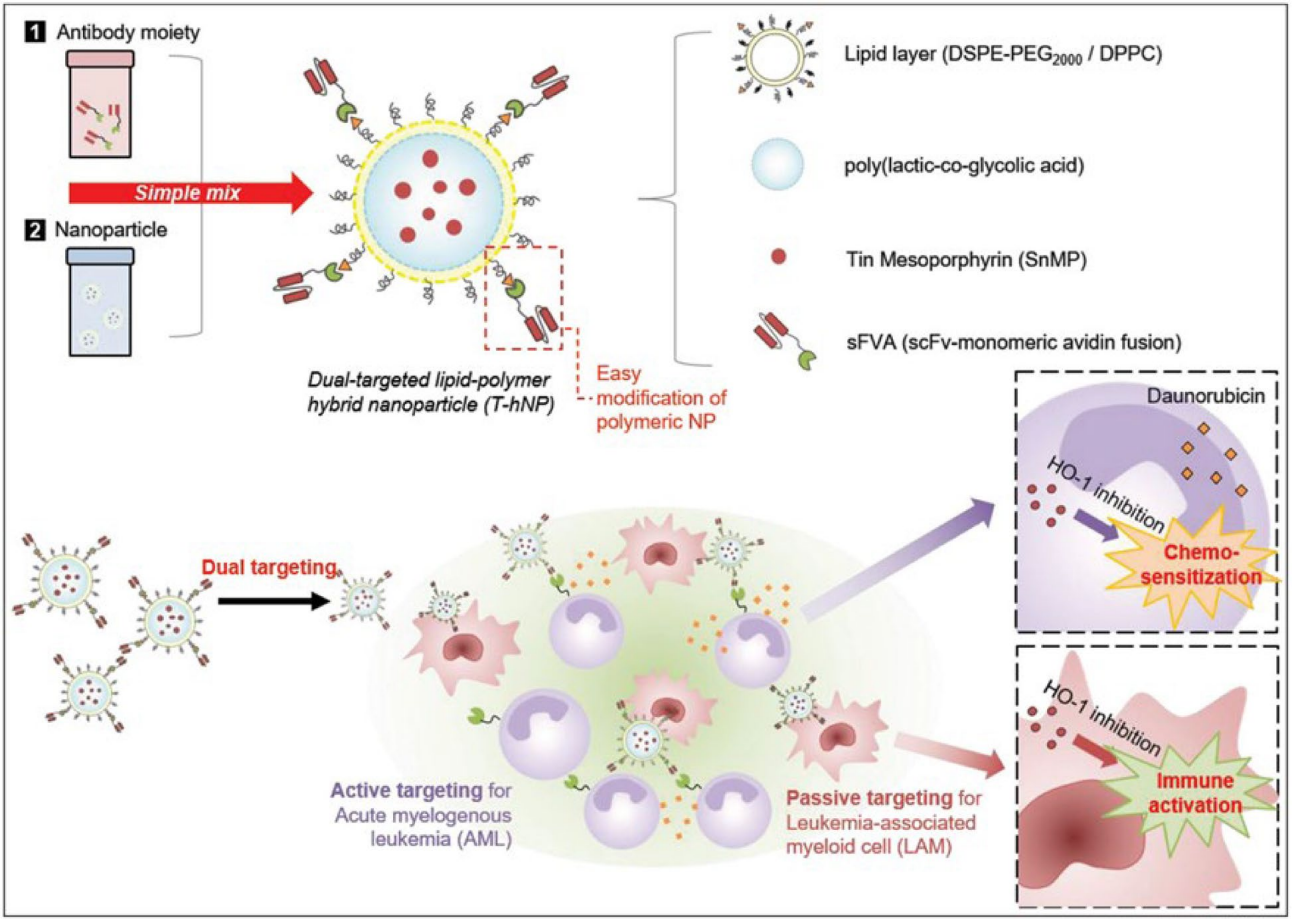
Representation of chemo-immuno-responsive effect of HO1-inhibiting hybrid nanoparticle against acute myeloid leukemia (AML). The nanoparticles show active targeting effect upon sFVA modification while are passively targeted via electrostatic interaction with the cell membrane causing phagocytic uptake. The figure is reproduced under the terms of the Creative Commons CC BY license﻿. Copyright John Wiley and Sons [284]

Dai et al. (2018) created TF-decorated PTX-loaded LPHNPs (TPLN) with the objective of increasing chemotherapy effectiveness in leukemia cells. The authors observed a biphasic release pattern from the drug-loaded formulation. At the end of 24 h, about 30% of the drug was released from the NPs, which lasted until 75 h. TF-decorated PTX-loaded NPs had a greater targeting potency and graded lethal impact on HL-60 cancer cells than PTX-loaded NPs. Targeted and non-targeted NPs showed IC_50_ values of 0.45 and 2.8 µg/mL, respectively. TF-decorated PTX-loaded NPs showed a notable death of cancer cells. Overall, the results clearly demonstrated the potential of TF-decorated PTX-loaded LPHNPs formulations to target leukemia cells [282]. Working in a similar area, Yong et al. (2020) developed LPHNPs decorated with an HO1-inhibitor; tin mesoporphyrin [(SnMP), (antioxidant and cytoprotective enzyme)]. These LPHNPs were further functionalized with an engineered antibody for acute myelogenous leukaemia (AML) cancer immunotherapy (Fig. 16). When targeted NPs were injected intravenously into the human AML-bearing orthotopic mice model, NPs actively targeted human leukemia cells and passively targeted CD11b^+^ myeloid cells in the bone marrow location. By reprogramming bone marrow myeloid cells, the targeted NPs improved the chemotherapeutic impact of daunorubicin (cerubidine) and boosted immunological response. This showed that the monocyte lineage was established and that inflammatory genes were installed. Ex vivo research revealed that HO1-inhibited bone marrow CD11b + myeloid cells and had a stronger immune response against apoptotic leukemia cells. The authors suggested that by combining chemo-sensitization of AML cells with immunological stimulation of bone marrow myeloid cells, HO1-inhibiting dual cell-targeted LPHNPs have a great promise as a new treatment in AML [284].

#### Dual drug delivery

Targeted co-delivery of drugs via LPHNPs may have synergistic effect on anti-cancer activity and same was studied for leukemia treatment. HA modified, DOX, and gallic acid (GA) co-laden LPHNPs were prepared for the treatment of leukemia. The produced HA tethered DOX-GA-LPHNPs formulations had the greatest cytotoxicity and synergistic impact on DOX resistant human HL-60 promyelocytic leukemia cells, DOX resistant human K562 chronic myeloid leukemia cells when the DOX/GA ratio was 2/1. Further, in vivo investigations confirmed a decrease in tumor volume from 956 mm^3^ to 213 mm^3^ when HA attached DOX-GA-LPHNPs were used, with a 77.7% inhibition rate. Overall, the study showed that HA attached DOX and GA co-laden LPHNPs could be a valuable tool in the treatment of leukemia [283].

#### Nucleic acid delivery

The significance of nucleic acid-based therapies has been eloquently illustrated by the current success of mRNA-based COVID-19 vaccinations. The selectivity and effectiveness of mRNA-coded expression of protein still need to be improved, especially outside the realm of preventive immunization, in order to fully estimate the potential of mRNA. Although, in comparison to previously explored research on DNA related therapies, mRNA has shown several advantages as nuclear localization for the expression of proteins is not required and even do not amalgamate genome, which in turn reduce the carcinogenic risk. The hybrid of lipid and polymeric system allow versatile attributes of lipidic membrane with unique characteristics of polymers. Andretto and group developed the lipoplex comprising of liposomes and mRNA, and functionalized with the negatively charged hyaluronic acid (HA) via electrostatic interaction. The aim behind introduction of HA was to reduce to aggregation, refine stability of preparation and improve clearance. Also, a disulfide bond (SS) cleavable pH-activated lipid-like material (ssPalm) was also included in the mixture of lipid to improve the release of mRNA in the cytosol and promote endosomal escape. After assessing the stability, the hybrid system was subjected to cellular internalization assessment in human derived monocytes and THP-1 cells. As compared to rhodamine, the cellular internalization was more for those treated with the hybrid nanopreparation. The in vivo fate demonstrated mRNA-lipocomplex in spleen which preference for macrophage expression while being the source for immune cells [285].

### LPHNPs for targeted delivery of therapeutics to the brain tumor

Apart from the above-discussed applications, LPHNPs have also been used to carry drugs across the blood–brain barrier (BBB). A BBB is a vascular barrier of the blood vessels restricting the in and out movement of molecules, ions, and cells between the blood and the brain. This restriction limits the delivery of bioactives to the brain. NPs mediated delivery of therapeutics had achieved significant outcomes against brain related diseases and LPHNPs were also investigated for the same. For this, carbamazepine (CBZ) loaded LPHNPs were synthesized for targeting brain tumor via the intranasal route. They used three distinct polymers chitosan, stearic acid, and glyceryl monostearate, in varying ratios to process five LPHNPs formulations (HN1, HN2, HN3, HN4, and HN5) by microemulsification followed by ultrasonication. Particle sizes ranged from 78.88 to 790 nm along the five formulations. The entrapment effectiveness of all formulations was determined to be between 62.66 to 88.31%, with in vitro releases ranging from 40 to 72%. As a result of chitosan polymer and lipid in the same ratio, the HN1 formulation matched the Korsmeyer Peppas release pattern while drug release from HN2 and HN3 formulations followed the Higuchi paradigm. The drug release from HN2 and HN3 was dependent on the porosity and tortuosity of the lipid matrix, thus as the lipid content increased, the drug release reduced due to the decreasing influence of the porosity of the lipid matrix. The Korsmeyer-Peppas model best fitted the drug release from HN4 and HN5 formulations. This suggested that the larger concentration of hydrophilic polymer chitosan restricted drug release in both formulations. The ratio of AUC (Brain) to AUC (Plasma) was reported to be 0.7144 in a pharmacokinetic study of CBZ. This translates to a slightly equal distribution of drug into the brain (target) and plasma (non-target). The C_max_ in the brain, on the other hand, was reached in less than 5 min and was reported to be 3230 ng. Although, in plasma, it took roughly 30 min to reach C_max_, which was reported to be 1298 ng. The AUC (Brain) to AUC (Plasma) C_max_ ratio was determined to be 2.996, indicating that the brain had a greater concentration of CBZ than plasma. The drug targeting efficiency (DTE) was discovered to be 3.698. The investigation found that the HN5 formulation had the greatest concentration in the brain and had the maximum drug targeting efficacy due to the high chitosan ratio, thus demonstrating a viable approach to deliver drugs across BBB [286]. To study the glioma targeting disposition, researchers used FA as well as cRGDfK decorated and PTX conjugated LPHNPs. The produced LPHNPs were predicted to pass across the BBB with ease and then target glioma cells with high integrin levels. Compared to non-targeted LPHNPs formulations, FA and cRGDfK decorated PTX-LPHNPs showed much stronger in vitro cell uptake, inhibitory efficacy, and cell apoptosis. The results of the in vivo anti-tumor studies showed that the median survival time for Balb/c mice treated with FA as well as cRGDfK decorated PTX-LPHNPs (42 days) was significantly longer than free PTX (14 days), control group (12 days) and non-targeted PTX-LPHNPs. The investigations found that the dual-targeted PTX-LPHNPs could successfully cross the BBB and deliver much greater quantities of drug to brain tumor microenvironments, resulting in a better therapeutic response [287–290]. Further, to treat temozolomide (TMZ) resistant glioblastoma by gene therapy, clustered regularly interspaced short palindromic repeats (CRISPR)- associated protein 9 (CRISPR/Cas9) encapsulated plasmids targeting O6-methylguanine-DNA methyltransferase (MGMT), encapsulated LPHNPs was constructed. MGMT is responsible for TMZ resistance in glioblastoma. To pass the blood–brain barrier restriction, the NPs were combined with the microbubbles. The NPs were targeted with cRGD. The delivered formulation effectively down-regulated the target gene and increased the sensitivity of the TMZ towards T98G cell line. When exposed to the focused ultrasound, the targeted microbubble-LPHNPs effectively accumulate inside the tumor region of orthotopic tumor-bearing mice (NOD-SCID) and significantly inhibited the tumor growth [291–293]. Similarly, to improve brain delivery and avoid opsonization, researchers produced LPHNPs containing PEG-based surfactants (SAA), tocopherol PEG succinate (TPGS), or Solutol HS 15. The LPHNPs were loaded with flavonoid rutin (RU) which is Calendula officinalis L. flower extract and has been proved as a likely anti-Alzheimer agent. With mean residence times of 1.90, 2.13, and 3.04 h, all loaded LPHNPs had a short resident duration (RU). Meanwhile, Tween, TPGS, and Solutol-based LPHNPs formulations showed a substantial increase in RU bioavailability (*p* < 0.05) of around 160-fold, 98-fold, and 159-fold, respectively. These findings revealed the structural uniqueness of the developed formulation due to the presence of PEG moieties, which provide a triggered stealth effect, and a low level of macrophage identification and thus a relatively long circulation property to NPs. LPHNPs that had previously been sheathed with Solutol had the highest peak plasma level, followed by Tween, and finally TPGS. Solutol-LPHNPs had a C_max_ that was 2.3 times greater than Tween-coated NPs. Tween and Solutol-based LPHNPs, on the other hand, showed enhanced total systemic availability and comparable bioavailability, but TPGS-LPHNPs had a considerably poorer bioavailability, as seen by lower AUCs. As a result, the biodistribution characteristics were used to investigate the in vivo research. Biodistribution investigations revealed no significant variations in RU accumulation inside the brain. Although, phagocytic uptake differed across various LPHNPs formulations. TPGS-LPHNPs had a larger drug assemblage in RES organs: liver > kidney > spleen, compared to Tween encased particles. This study showed that PEG-SAA could successfully modify LPHNPs and it can be used to deliver TPGS and Solutol to the brain in a targeted manner [294]. Various studies of brain targeting via LPHNPs are summarized in Table 8.

**Table 8:** Application of LPHNPs in the treatment of brain tumors

Sr. No	Lipid component	Polymer component	Targeting moiety	Drug	In vitro	In vivo	Ref
**1**	Glyceryl mono stearate, Stearic acid	Chitosan	_	Carbamazepine	_	Wister rats	[286]
**2**	DSPE-PEG-2000, DPPC, cholesterol	PLGA	cRGD	CRISPR/Cas9	T98G cells	NOD-SCID mice	[291]
**3**	Lecithin, Soybean phosphatidylcholine,	PLGA	Tocopherol	Rutin (Flavanoid)	_	Swiss mice	[294]

## Toxicity issues and challenges

With NPs and liposomal delivery, there are several difficulties and toxicity concerns. The particle size of NPs plays an essential role in drug delivery technology. As, NPs are smaller and have less mass, they have a greater specific surface area, which promotes interaction with biological components such as fats, nucleic acids, carbohydrates, fatty acids, and proteins along with variety of undesirable metabolites. Systemic administration of the nanoparticles majorly affected by mononuclear phagocytic system (MPS) and helps in the clearance of the nanoparticles via a phagocytes, including monocytes, macrophages, and dendritic cells, in all organs, especially the spleen, liver and lymph nodes which contain resident macrophages [295–297]. MPS begin with the opsonization facilitated by adsorption of opsonins such as immunoglobulins, complement proteins, and fibrinogen on the surface of nanoparticles and then engulfment by macrophages [298]. To avoid the interaction between the nanoparticles and MPS, nanoparticle surface modification has been carried out(surface coating using proteins, polymers, and cell membranes). Change in the parameters like shapes, sizes, and chemical compositions of nanoparticles also inhibit clearance of the nanoparticles by MPS [299, 300]. Salvador-Morales et al., 2009, studied activation of complement system by amine, carboxyl and methoxyl terminated LPHNPs prepared with a hydrophilic poly (ethylene glycol) (PEG) shell, PLGA core and a soybean phosphatidylcholine (lecithin) monolayer at the interface of the inner and outer layer. Here, amine functionalized LPHNPs more effectively activate the complement system than other NPs. But none of the three NP formulations significantly stimulate the complement system compared to Zymosan (positive control), a well-known alternate pathway complements system activator, whereas all three slightly activate the complement system more than human serum (negative control). The serum amyloid A-4 protein precursor preferred to bind to NPs with surface amine and/or methoxyl groups, according to an experiment with the binding of LPHNPs over plasma protein and human serum protein. Methoxyl groups terminated LPHNPs did not showed any effect on clotting time in coagulation studies [301]. The reduced size also makes it easier for bioactive to enter the tumorous microenvironment of the cell, causing cellular damage as well as the buildup of metallic NPs. In vivo toxicity, lung inflammation, systemic irritation, platelet activation, increased heart rate variability, and vasomotor dysfunction are all possible consequences of smaller particle size [302]. Other issues such as oxidative stress and intracellular calcium homeostasis are fundamentally affected by NPs delivery, resulting in cell damage, death, and cell cycle dysregulation [303]. The lipid-based NPs formulations have been reported for toxicity issues with two major organs, the liver and spleen, associated with its distribution and metabolism. In addition, other studies have also reported that high doses of the NPs is also one of the major reasons for toxicity because of the accumulation of the high contents of the lipid in liver and spleen [304, 305]. Exposure to NPs has been linked to a number of pathological disorders, including respiratory, cardiovascular, lymphatic, autoimmune, neurodegenerative, and cancer disorders, with malignancies developing years later. Similarly, in the case of liposomes, phospholipids are the main components, and changes in stability or kinetics might result in an increase in hazardous potency, particularly when liposomes are administered parenterally. Following systemic injection of liposomes, the RES is the main location of liposome assembly. The innate immune system, which includes RES cells, has generated concerns about liposome saturation of macrophages, which causes immunosuppression and raises the risk of infection [306–308]. LPHNPs are the most promising option for overcoming the toxicity problems and problems associated with individual carrier system delivery. The right combination of lipids and polymers can achieve improved physicochemical properties of hybrid NPs such as size, surface area, particle charge, drug encapsulation, drug deposition, accumulation, improvement of physical stability drug release modulation, and acceleration of cellular uptake [95, 307, 308].

## Conclusion and future perspective

LPHNPs are a type of cutting-edge innovation in biomedical industry and cancer that combines the benefits of many nanoparticles into a single solution. With an aim to achieve large-scale, long-term advantages from intelligence technology, these nanostructures came into the limelight. LPHNPs are a particularly appealing delivery vehicle for cancer treatments due to their high loading potential for various active moieties, superior bloodstream stability, and cargo-delivering potential in vivo. Due to adjustable drug release profile, targeting potential, enhance bioavailability, cellular accumulation, ease of synthesis and stability provided by PEG, makes them a step ahead for cancer therapy. All of these characteristics point to LPHNPs remarkable promise as flexible carrier and enhanced therapeutic potential for cancer treatment. LPHNPs have also been entitled for their effectiveness in translation of novel clinical and drug delivery concerns from laboratory to bedside, with a major and long-term impact on cancer therapy. LPHNPs come in a variety of configurations and have triggered release properties as well as long-term in vitro and in vivo behavior. Such a hybridize arrangement is capable of delivering the optimal quantity of drug to a very precise spot while causing minimal adverse effects to the other normal cells. It can perform dual drug therapy with synergistic effects, and the addition of internal or external stimuli makes this opportunistic carrier system more helpful and sophisticated. The goal of diagnosis can be met simultaneously with the perception of theranostics. With LHPNPs, we were able to forecast and maintain high expectations in the research sector, as well as in industrial production and scalability. LPHNPs have the ability to serve an enormous range of applications in the future in addition to translational prospects for prospective clinical studies. It will be in charge of increasing cancer patients' life expectancy and improving their quality of life in the future. The lipid hybrid nano-system should enter into the clinical market with special emphasis on increasing the life expectancy. Not limiting to cancer therapy, LPHNPs applicability should also be explored in various other life threatening disease as well.

## Data Availability

Not applicable.
